# Toward Robust Cognitive 3D Brain-Inspired Cross-Paradigm System

**DOI:** 10.3389/fnins.2021.690208

**Published:** 2021-06-25

**Authors:** Abderazek Ben Abdallah, Khanh N. Dang

**Affiliations:** ^1^Adaptive Systems Laboratory, Graduate School of Computer Science and Engineering, The University of Aizu, Aizu-Wakamatsu, Japan; ^2^VNU Key Laboratory for Smart Integrated Systems (SISLAB), VNU University of Engineering and Technology, Vietnam National University, Hanoi, Vietnam

**Keywords:** spiking neural network, neuromorphic, 3D-ICs, fault-tolerance, mapping algorithm

## Abstract

Spiking Neuromorphic systems have been introduced as promising platforms for energy-efficient spiking neural network (SNNs) execution. SNNs incorporate neuronal and synaptic states in addition to the variant time scale into their computational model. Since each neuron in these networks is connected to many others, high bandwidth is required. Moreover, since the spike times are used to encode information in SNN, a precise communication latency is also needed, although SNN is tolerant to the spike delay variation in some limits when it is seen as a whole. The two-dimensional packet-switched network-on-chip was proposed as a solution to provide a scalable interconnect fabric in large-scale spike-based neural networks. The 3D-ICs have also attracted a lot of attention as a potential solution to resolve the interconnect bottleneck. Combining these two emerging technologies provides a new horizon for IC design to satisfy the high requirements of low power and small footprint in emerging AI applications. Moreover, although fault-tolerance is a natural feature of biological systems, integrating many computation and memory units into neuromorphic chips confronts the reliability issue, where a defective part can affect the overall system's performance. This paper presents the design and simulation of R-NASH-a reliable three-dimensional digital neuromorphic system geared explicitly toward the 3D-ICs biological brain's three-dimensional structure, where information in the network is represented by sparse patterns of spike timing and learning is based on the local spike-timing-dependent-plasticity rule. Our platform enables high integration density and small spike delay of spiking networks and features a scalable design. R-NASH is a design based on the Through-Silicon-Via technology, facilitating spiking neural network implementation on clustered neurons based on Network-on-Chip. We provide a memory interface with the host CPU, allowing for online training and inference of spiking neural networks. Moreover, R-NASH supports fault recovery with graceful performance degradation.

## 1. Introduction

The brain-inspired computing paradigm takes inspiration from the biological brain to develop energy-efficient computing systems for future information processing capable of efficiently executing highly complicated tasks, such as decision-making and perception. Spiking neural networks (SNNs) attempt to mimic the information processing in the mammalian brain based on parallel arrays of neurons that communicate via spike events. Different from the typical multi-layer perceptron networks, where neurons fire at each propagation cycle, the neurons in SNN model fire only when a membrane potential reaches a specific value. In SNN, information is encoded using various encoding schemes, such as coincidence coding, rate coding, or temporal coding (Levin et al., 2014). SNN typically employs the integrate-and-fire neuron model in which a neuron generates voltage spikes (roughly 1 ms in duration per spike) that can travel down nerve fibers if they receive enough stimuli from other neurons with the presence of external stimuli. These pulses may vary in amplitude, shape, and duration, but they are generally treated as identical events. To better model the dynamics of the ion channel in a biological neuron, which is nonlinear and stochastic, the Hodgkin-Huxley (Goldwyn et al., 2011) conductance-based neuron is often used. However, the Hodgkin-Huxley model is too complicated to be used for a large-scale simulation or hardware implementation.

Software simulation of SNN (Hazan et al., 2018; Stimberg et al., 2019) is a flexible method for investigating the behavior of neuronal systems. However, simulation of a large (deep) SNN system in software is slow and cannot fully exploit the overall system performance. An alternative approach is a hardware implementation, which provides the possibility to generate independent spikes accurately and simultaneously output spikes in real time. Hardware implementations of SNNs (neuromorphic) also have the advantage of computational speedup over software simulations and can take full advantage of their inherent parallelism. Specialized hardware architectures with multiple neuro-cores could exploit the parallelism inherent within neural networks to provide high processing speeds with low power, which make SNNs suitable for embedded neuromorphic devices and control applications (Vu et al., 2019). In general, the neuromorphic hardware systems consist of multiple nodes (or clusters of neurons) connected via an on-chip communication infrastructure (Akopyan et al., 2015; Ogbodo et al., 2020). Expansion using a multi-chip system and off-chip interconnects is also a viable solution for scaling up SNNs (Akopyan et al., 2015; Davies et al., 2018). In recent years, integrating many neurons on a single chip while providing efficient and accurate learning has been investigated (Schemmel et al., 2010; Benjamin et al., 2014; Furber et al., 2014; Akopyan et al., 2015; Davies et al., 2018).

The challenges that need to be solved toward designing an efficient neuromorphic system include building a small-size, parallel, and reconfigurable architecture with low-power consumption, an efficient neuro-coding scheme, and an on-chip learning capability. Moreover, since the number of neurons to be connected is at least 10^3^ times larger than the amount of PEs (Processing Elements) that need to be interconnected on modern multicore/multiprocessor SoC platforms (Furber, 2016), the on-chip communication and routing network is another major challenge. In a modern deep neural network (DNN) design, one neural network layer is often a 2D structure. However, the “mimicked” network is generally a 3D structure. Therefore, mapping a 3D structure onto 2D circuits may result in either multiple long wires between layers or congestion points (Vu et al., 2019; Dang et al., 2020b; Ikechukwu et al., 2021).

An event-driven neuromorphic system relies on the arrival of spikes (action potentials) to compute (Purves et al., 2008). Therefore, the arrival times of action potentials are critical to allow accurate and consistent outputs. Since the shared bus is no longer suitable for multicore systems and point-to-point interconnects cannot serve a high fanout wires (Lee et al., 2008), moving to a new on-chip communication paradigm with the ability to extend to multiple-chip interconnects is needed. One of the consensuses of state-of-the-art architecture is to utilize the parallelism and scalability of 2D Network-on-Chip (NoCs) (Akopyan et al., 2015; Davies et al., 2018) and further extend it to multichip systems. In this approach, the neurons of the silicon brain are clustered into nodes that are attached to micro-routers.

**Figure 1:**
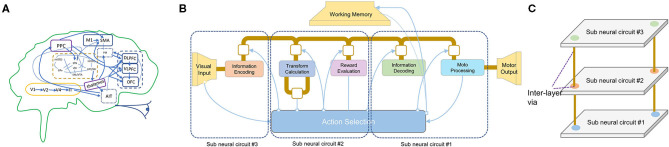
Neuro-inspired 3D silicon brain. **(A)** The anatomical architecture of Spaun indicates major brain structures and their connectivity. **(B)** The architecture of Spaun, where thick dark-yellow lines illustrate communication between elements of the cortex while thin lines show connectivity between Basal Ganglia and the cortex. **(C)** A possible mapping of a Spaun system into 3D-IC.

From another hand, semiconductor development is confronting the end of Moore's Law, which no longer allows us to reduce the feature size as we reach the atomic scale. To get to the “More than Moore” goal (Waldrop, 2016), heterogeneous integration is a suitable approach to integrate more transistors in the same die. One of the popular approaches is to stack the conventional 2D wafers together to form a 3D-chip (Banerjee et al., 2001). Another method is monolithic 3D-ICs that support multiple silicon layers based on small vias (Panth et al., 2014). The Through-Silicon Vias (TSVs) or Monolithic Intertier Vias (MIVs) constitute one of the main interlayer communication mediums. The 3D-Network-on-Chip (3D-NoC) (Ben Ahmed and Ben Abdallah, 2013) is also a promising approach that can further enhance the parallelism and scalability of multicore and neuromorphic systems. Figure 1 illustrates a potential mapping of an emulated silicon brain into 3D-ICs. Here, the anatomical architecture of Spaun indicates large brain structures, and their connectivity is illustrated with thick dark-yellow lines for communication between elements of the cortex. In contrast, thin lines show connectivity between Basal Ganglia and the cortex (Eliasmith et al., 2012; Vu et al., 2019). However, despite bringing several benefits of lower power, smaller footprints, and low latency, the integration of a neuromorphic system into 3D-ICs was not well investigated.

This paper presents a reliable three-dimensional digital neuromorphic system, named R-NASH, geared explicitly toward the 3D-ICs biological brain's three-dimensional structure, where information in the network is represented by sparse patterns of spike timing and learning is based on the local spike timing-dependent plasticity rule. R-NASH is based on the Through-Silicon-Via technology, facilitating the spiking neural network implementation on clustered neurons based on Network-on-Chip. Morever, R-NASH features efficient spiking neuron mapping algorithms to map the neurons into suitable R-NASH clusters based on a genetic algorithm (GA). Furthermore, R-NASH supports faults recovery with graceful performance degradation.

The rest of this paper is organized as follows: section 2 presents related works. section 3 describes the proposed R-NASH platform and presents the neuron mapping method based a graph-based algorithm. section 4 presents the evaluation results. Finally, section 5 concludes this paper.

## 2. Related Works

In this section, we summarize the related works on neuromorphic systems. One of the most crucial computing units for SNN is neuron architecture. Due to its low complexity, Leaky-Integrate-and-Fire (LIF) or Integrated-and-Fire (IF) have been the de facto choice for hardware-based neuromorphic systems (Benjamin et al., 2014; Akopyan et al., 2015; Davies et al., 2018; Frenkel et al., 2018; Stimberg et al., 2019). However, there are several variations in the neuron design. While mixed-analog-digital neurons (Benjamin et al., 2014; Stimberg et al., 2019) can only support a certain level of computing due to their primitive design, full digital neurons can perform more complex computation with the trade-off of larger area cost. Chen et al. (2018) use relative leak on the current membrane potential instead of constant and added bias on input. Work in Diehl and Cook (2015) (software) uses adaptive thresholds that decaying overtime during learning. These variations may affect the design's area cost and timing; however, they all follow the same principle as above. The work in Frenkel et al. (2018) and Frenkel et al. (2019) supports loading neuron parameters, such as membrane potential, threshold, and so on.

Another major problem is the neuron communication approach. Point-to-point or bus-based interconnects cannot scale up to a large system (i.e., a million neurons and a billion synapses). Therefore, adopting an on-chip and off-chip network has been a consensus among state-of-the-art works (Benjamin et al., 2014; Akopyan et al., 2015; Davies et al., 2018; Frenkel et al., 2018; Stimberg et al., 2019).

*Neurogrid* by Stanford University is one of the early works on simulating biological brains in real-time with millions of neurons (Benjamin et al., 2014). Neurogrid is based on mixed-analog-digital neuron design, where the neuron is based on a capacitor that can be charged by incoming action potentials (or spikes). The voltage of the capacitor is further put to a voltage comparator to compare with the threshold voltage. Once the comparator outputs “1,” which means the capacitor's voltage has crossed the threshold, the outcome is sent as the action potential, and the capacitor is reset to ground voltage. The operation of the neuron is approximately closed to the Integrate and Fire model. The output is packetized with variable length flits to send to an on-chip and off-chip tree-based communication fabric that supports multi-casting (Benjamin et al., 2014). To allow a better scalable design, Neurogrid follows an Address Event Representation (AER), in which the addresses of their sources represent spikes.

The next notable work is BrainScaleS (Schemmel et al., 2010; Scholze et al., 2011) which is built with mixed-analog-digital neurons. The structure of BrainScaleS's neurons can model the Leaky-Integrate-and-Fire as it supports the leaky function. Communication of BrainScaleS is based on a so-called hierarchical digital routing infrastructure. The on-chip and off-chip communication also follow the AER protocol.

To support more complex neuron computation, SpiNNaker (Furber et al., 2014) uses one million homogeneous ARM968 processors for emulation. Each ARM processor can simulate a thousand neurons that allow the system to save up to a billion neurons. SpiNNaker uses a fully packetized communication infrastructure built on a folded two-dimensional toroidal mesh where each node can communicate with six neighbors. The SpiNNaker system is also based on the AER format with table-based multi-casting support.

TrueNorth (Akopyan et al., 2015) and Loihi (Davies et al., 2018) are the two fully digital neuromorphic chips designed by IBM and Intel, respectively. Both TrueNorth and Loihi chips are based on two-dimensional mesh topology interconnects, which allow both on-chip and off-chip communications. While TrueNorth has fixed LIF neurons, Loihi enables users to program the neuron operation, supporting more detailed neural computation. Unlike volatile analog neurons, digital neurons can be stored and reloaded from memory. Therefore, both TrueNorth and Loihi adopt the same fashion, allowing them to use one physical neuron to emulate multiple ones. Although the performance is not like in parallel neuron method, the current ASIC high frequency still enables them to operate close to the biological speed. Due to their low-cost constraints, none of these works support multicast, requiring them to utilize the unicast approach.

Thanks to Network-on-Chips' scalability and parallelism, most of the state-of-the-art works have adopted one form of on-chip/ff-chip communication. However, scaling using 3D-ICs has not been well considered in previous neuromorphic chip designs. For Deep Neural Networks, Joseph et al. (2021) claimed that 3D architectures could speedup 9.14× when compared to the 2D ones. Instead of using a 2D array of Multiple-Accumulation-Units (MACs), the authors converted to the 3D structure by using either through-silicon-vias (TSVs) or monolithic intertier vias (MIVs). Previously, we have explored the ability to integrate SNN into 3D-NoCs (Dang and Ben Abdallah, 2019; Ikechukwu et al., 2021). Instead of using 2D-NoCs, we extend the NoC to the third dimension, allowing the conventional neuron cluster design to work with small changes in the routing mechanism. As most of the state-of-the-art works still focus on dealing with 2D-ICs neuromorphic systems, we observe that exploring to 3D-ICs approach can bring several benefits such as smaller footprints and small number of hops.

## 3. R-NASH Platform Design

**Figure 2:**
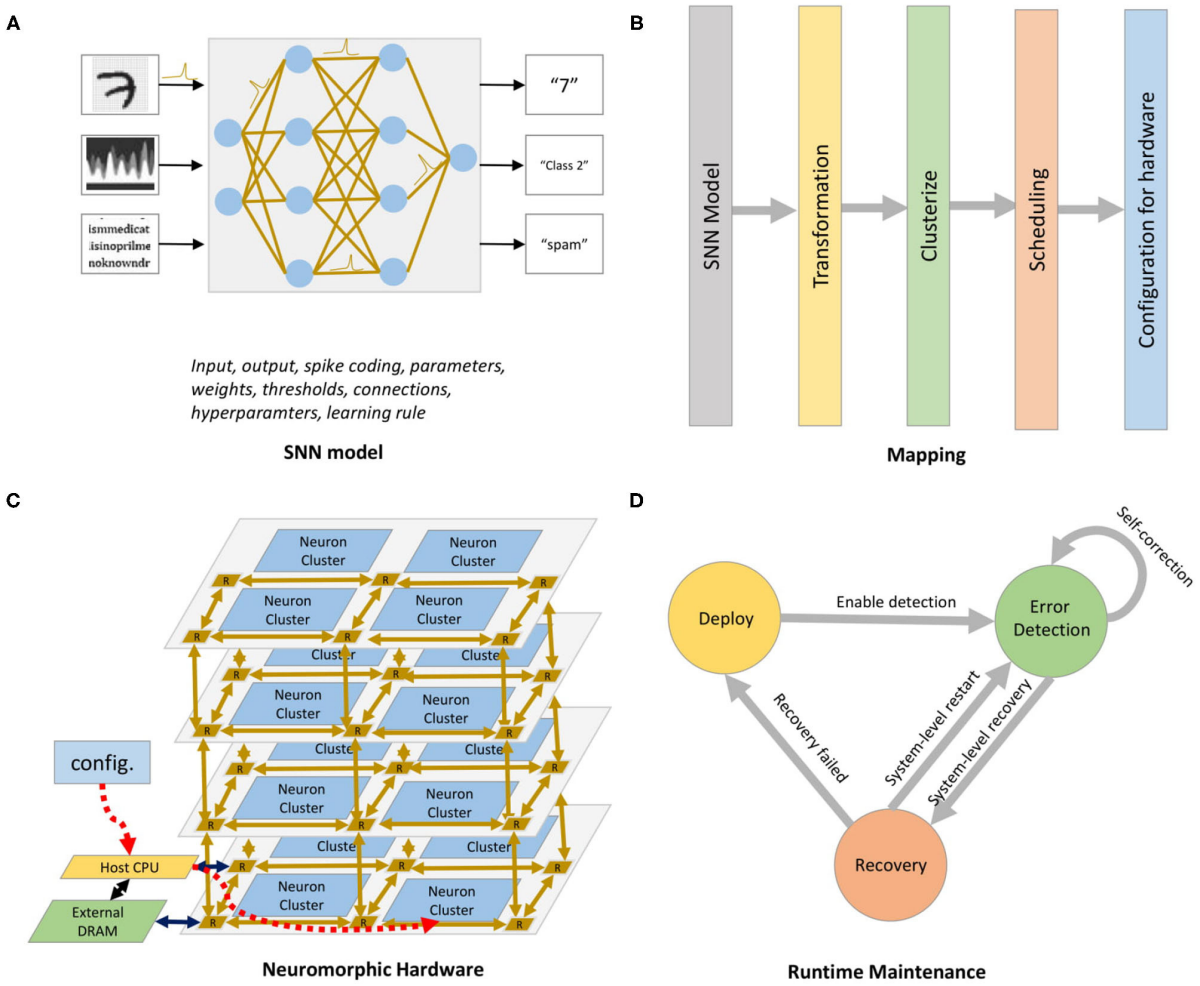
R-NASH platform. **(A)** SNN model. **(B)** Mapping into the neuromorphic hardware. **(C)** R-NASH hardware. **(D)** Run-time maintenance.

The R-NASH platform design, shown in Figure 2, consists of four phases. First, the software spiking neural network *model* is developed with various configurable parameters, including neurons, synapses, thresholds, learning types, and interconnects. Then, the *neurons mapping* phase is performed based on four main steps: (1) Transformation: convert the hardware parameters to values that can be read and executed by the neuromorphic hardware, (2) Clusterize: group and find the suitable mapping of neurons, (3) Scheduling, and (4) Configuration. We use a genetic algorithm (discussed later) to optimize the flow. As a highly complex design, neuromorphic hardware is generally prone to soft and permanent faults, leading to performance degradation. Therefore, the *run-time maintenance* stage checks and recovers from faults.

### 3.1. R-NASH Hardware Building Blocks

**Figure 3:**
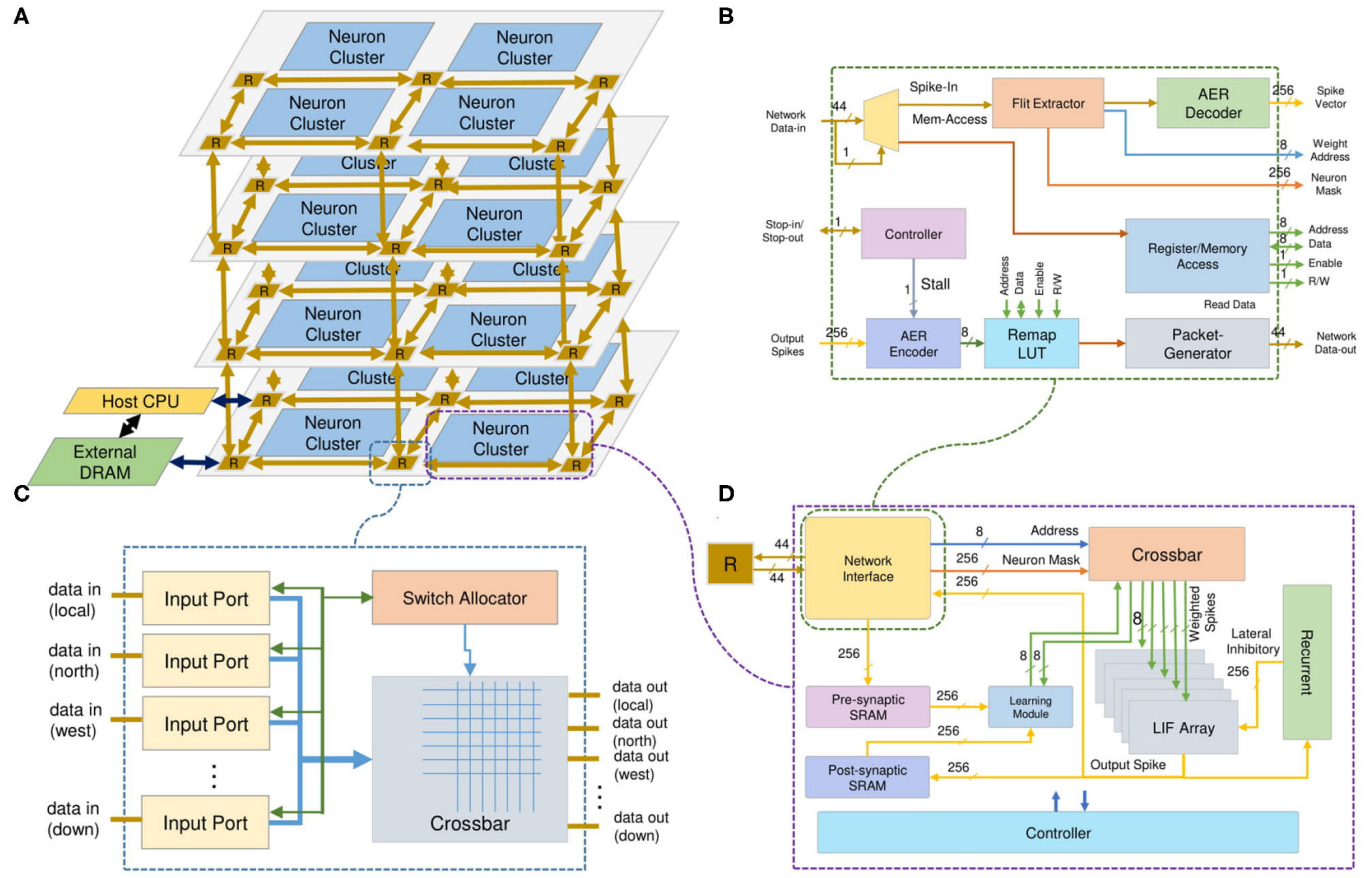
R-NASH architecture. **(A)** Neuromorphic System. **(B)** Network-Interface. **(C)** 3D-Router. **(D)** SNPC Core.

The overall architecture of the neuromorphic system, shown in Figure 3, is based on a 3D-IC approach to model the three-dimensional structure of the brain. The neurons and their synapses are grouped into neuron clusters or nodes. Instead of using a point-to-point neuron connection, we use a packet-switched mesh-based 3D-Network-on-Chip architecture. The inter-layer communication medium is Through-Silicon-Via (Dang et al., 2020a), one of the advanced technologies for stacking 3D-ICs. While a 3D-Mesh NoC handles the communication, the computation is done by neuron clusters (nodes) as shown in Figure 3D. The incoming spikes in AER (Address-Event-Representation) protocol are stored in memory and decoded to obtain the address and the read enable signal for the weight memory. By reading the synapses from memory, the system emulates the weighted spikes for LIF neuron inputs. After receiving the address of the corresponding synapse and the *enable* signal, a series of weighted inputs are sent to the dedicated LIF neuron, which accumulates the value, subtracts the leak, and checks the firing condition. The output spike is finally stored in a postsynaptic SRAM and sent to the Network-on-Chip. More details about the neuron clusters and the 3D NoC router architectures are given in sections 3.1.1, 3.1.3, respectively.

#### 3.1.1. Spiking Neural Processing Core (SNPC)

Figure 3D shows the architecture of the SNPC, which is the backbone of the proposed neuromorphic system. The SNPC consists of four major modules: (1) *Network Interface*, which supports the communication via our 3D NoC; (2) *Crossbar*, which supports the spikes decoding phase and extracts the corresponding weights; (3) *LIF array*, which consists of multiple LIF neurons functioning in a parallel; and (4) *STDP learning module*.

Once a packet is fed to the network interface from the 3D-NoC router, its information is decoded to decide the packet type. There are two types of packets: (1) *spikes* (or action potentials) and (2) *memory access* (read/write in single/burst). For the spike flits under the AER format, the NI decodes the equivalent address in the memory and sends it to the crossbar to obtain the weight. Once a neuron of the the SNPC fires, its index or address is encoded and sent to the 3D-NoC to emulate inter-neuron communication. For memory access, the NI provides an interface to read and write each neuron's weight memory and parameters in single and burst transaction mode.

**Figure 4:**
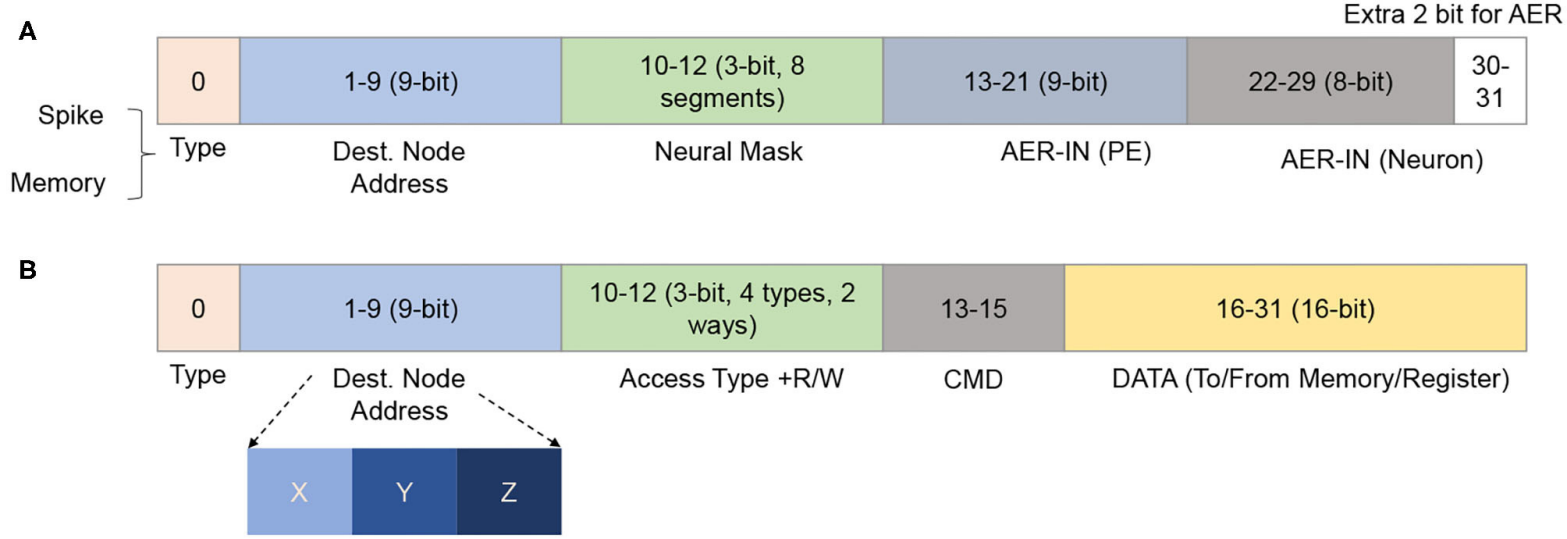
R-NASH flit formats. **(A)** AER flit format. **(B)** Memory access flit format.

The *Network Interface* allows the neurons to communicate via the on-chip network infrastructure. The spike (in AER format) flit is converted to the address of the weight SRAM. A flit provides the instruction and the required addresses to read/write to/from the memory cells and registers in the neuron cluster. Here, the memory access flits are issued by a master (or external host) processor in the system. The NI supports two types of read/write commands: single and burst. The individual read/write only provides access to one element per request, while an argument of length must follow the burst ones. The NI converts the requested address to the local address of each weight memory or LIF array. Figure 4 illustrates R-NASH's flits. The first bit indicates whether the flit is a spike (0) or memory access (1). With the spike flit, it is followed by four fields: (1) destination node address (9-bit), (2) neuron mask to allow the sparse connection (3-bit: 8 types of sparse), (3) AER of the source node (9-bit) and (4) AER of the neuron in the source node. Here, the AER of a firing neuron is represented via two fields: node address and neuron address; this allows the system to scale up to 8 × 8 × 8 3D-NoC nodes (512 nodes) and 256 neurons/node. Since we only use 30 in 32-bit 3D-NoC flit, we can extend the neuron ID field to 10-bit to allow indexing 1,024 neurons/node, allowing the R-NASH system to have 0.5 million neurons and 0.5 billion synapses. Large-scale configuration can extend the field to support more bits in neuron ID, PE ID, or NoC address.

For memory access spikes, there are four types: (1) single read, (2) burst read, (3) single write, and (4) burst write. The two-bit command field allows the system to inform the slave node to understand whether the transaction is done, kept, corrupted (need to rewrite/reread), or canceled. Since R-NASH is byte-addressable, the command field is followed by the address of memory/registers on the R-NASH node (16-bit). With the single read, the NI sends the data corresponding to the host node's address. With the burst read and write, the following flit consists of the length of these transactions.

Figure 3B shows the block diagram of the Network Interface (NI). The input spikes are classified into either input spikes or memory accesses. With the memory accesses, the NI provides an interface to read and write the data in all registers and memory blocks of the node. The read instruction makes the NI return the master processor value of the requested address. With the network's input spike, the NI decode phase gets the weight SRAM address and feeds it to the weight memory. For multi-layer SNNs or sparsity connections, the *Flit Extractor* provides the read enable (RE) signal for different layers or different links used in the weight memory. As a result, a node can have multiple AERs at the same address but for other neurons. The LIF array's output spike is fed into the AER decoder, which extracts the address of bit one (firing neuron). This address is then serially sent to the remap Look-Up-Table (LUT) to obtain the AER value of the receiving nodes.

As explained in the previous subsection, the input spikes (series of events determined by their timestamp and their polarity) are decoded to the weight address and neuron mask (read-enable signal) and fed to the crossbar. The crossbar is a set of SRAMs where each SRAM stores all synapses associated with a single neuron. The neuron mask signal is used to discard the unused weighted spike. After getting the address and the enable signal, the crossbar reads the synapses from the memory and sends them to the LIF array.

**Figure 5:**
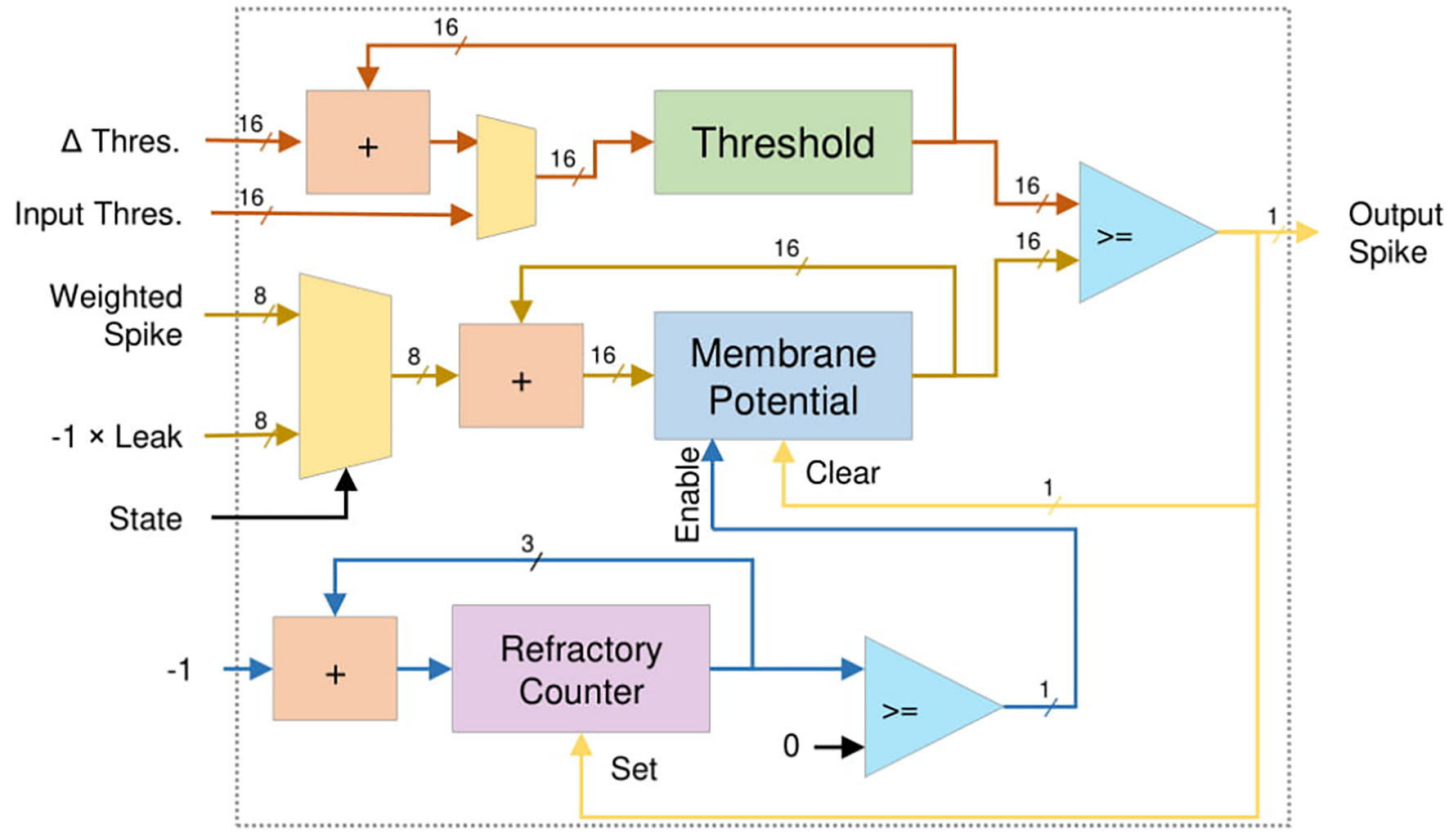
Temporal lif neuron model architecture.

To exploit the temporal correlation, the Leaky-Integrate-and-Fire (LIF) neuron model is selected for the proposed R-NASH architecture. Figure 5 shows the architecture of a LIF neuron. The weighted spike inputs (i_wspike) are fed into an adder and register structure for accumulation. At the end of each time step, the leak's inverted value is also fed to the adder to reduce the membrane potential. The neuron firing condition is then validated by confirming that the membrane potential has exceeded the neuron firing threshold. After the neuron fires, it sets the refractory countdown and stops working until the countdown is over. The period of this countdown is the refractory period. Theoretically, a LIF or IF computation is expressed with the the equation below:

(1) $$\begin{array}{r} V_{j}\left(t\right)=V_{j}\left(t-1\right)+\underset{i}{\sum }w_{i,j}\times x_{i}\left(t-1\right)-\lambda \end{array}$$

Where, *V*_*j*_(*t*) is the membrane potential of neuron *j* at time step *t*, *w*_*i,j*_ is the synapse weight between presynaptic neuron *i* and postsynaptic neuron *j*, *x*_*i*_(*t* − 1) is the output spike of presynaptic neuron *i*, and λ is the leak constant (λ = 0 for IF). The output of the neuron *j* is described by the equation bellow:

(2) $$x_{j}(t)=\{\begin{array}{cc} 1, & \;\text{if }V_{j}(t)\geq V\\ 0, & \;\text{otherwise} \end{array}$$

At the crossbar, the input spikes are multiplied with the weights to have weighted inputs. These weighted inputs are accumulated as the membrane potential, and when the accumulated value exceeds the threshold, a spike is fired. The memory module handles the multiplication of the input spikes and the weight of synapses. Since the input spike is binary, there is no actual multiplication hardware. Therefore, a simple register and adder is used to perform the accumulation.

#### 3.1.2. Controlling

There are two phases for controlling the R-NASH node: (1) *training* and (2) *inference*. If the *training* mode is enabled, the system enters the learning phases after each time step. If there is a postsynaptic spike of the emulated training time step, the weight is adjusted. Otherwise, the system skips to the next timestep. In the *inference* process, R-NASH starts with the synchronization of the timestep. Each node's registers are accessible via interfaces; therefore, the system can indicate, confirm, and change each node's timestep. Since the definition of timestep is loosely defined, it helps the LIF array to switch from the integration to the leaky phase. In other words, neurons can operate at different timesteps if needed. The operation of the SNPC follows four phases: (1) loading spikes, (2) integrating, (3) leak and firing, and (4) learning (if enabled). The first phase is for downloading spikes from presynaptic neurons via the interconnect. Due to the input cache's limited size, the spike is decoded and sent directly to the crossbar. On the other hand, it is packed to a spike vector for learning purposes due to its compactness in memory footprint.

#### 3.1.3. Fault-Tolerant Communication Network

R-NASH is based on a 3D-NoC that supports various fault-tolerance in input buffers, crossbar, routing hardware and pipeline (Dang et al., 2020b). The spike-based communication interconnect (3D-NoC) exploits temporal sparsity and consists of multiple routers (R) to handle the communication between the neuron custers (Dang and Ben Abdallah, 2019). Two types of flit are supported. The first type is the spike between neurons in AER format. The AER format flit is converted into the address of the weight SRAM. The second type of flit is memory access. To read and write to the memory cells and registers in the neuron cluster, a flit provides the instruction and the required arguments. Here, the memory access flits are issued by a master (or external host) processor in the system. We support two types of read/write commands: single and burst. The individual read/write only provides access to one element per request, while a lengthy argument must follow the burst ones. The NI converts the requested address to the local address of each weight memory or LIF array. The architecture of the NI is shown in Figure 3B.

The LIF array's output spike is fed into the AER decoder, which extracts the address of one bit (firing neuron). This address is then serially sent to the remap Look-Up-Table (LUT) to obtain the AER value in the receiving nodes.

### 3.2. R-NASH Learning

R-NASH system supports two learning methods: (1) off-chip learning based on a straightforward approach to load the parameters of a pre-trained ANN and map them to an equivalent accurate SNN, and (2) online learning based on STDP approach.

For off-chip learning, we adopt the method in Diehl et al. (2015). The feed-forward neural network is a fully connected model with a RELU (rectified linear units) activation function. It is trained as usual using back-propagation with zero bias throughout the training. When the training is complete, we map the network's RELU weights to the IF (Integrate and Fire) network. After that, the weights are normalized and converted to SNN. Finally, the converted weights are mapped to our R-NASH model to perform inference. Note that there is no refractory and leaky used in this conversion. After being normalized, the weights are quantized into a fixed bit format and loaded to the R-NASH system via a host CPU. In particular, we use 8-bit as the de-facto format in our system. However, adopting a smaller bit-width makes it possible to reduce the overall area cost because memories take up a significant portion of the system.

**Figure 6:**
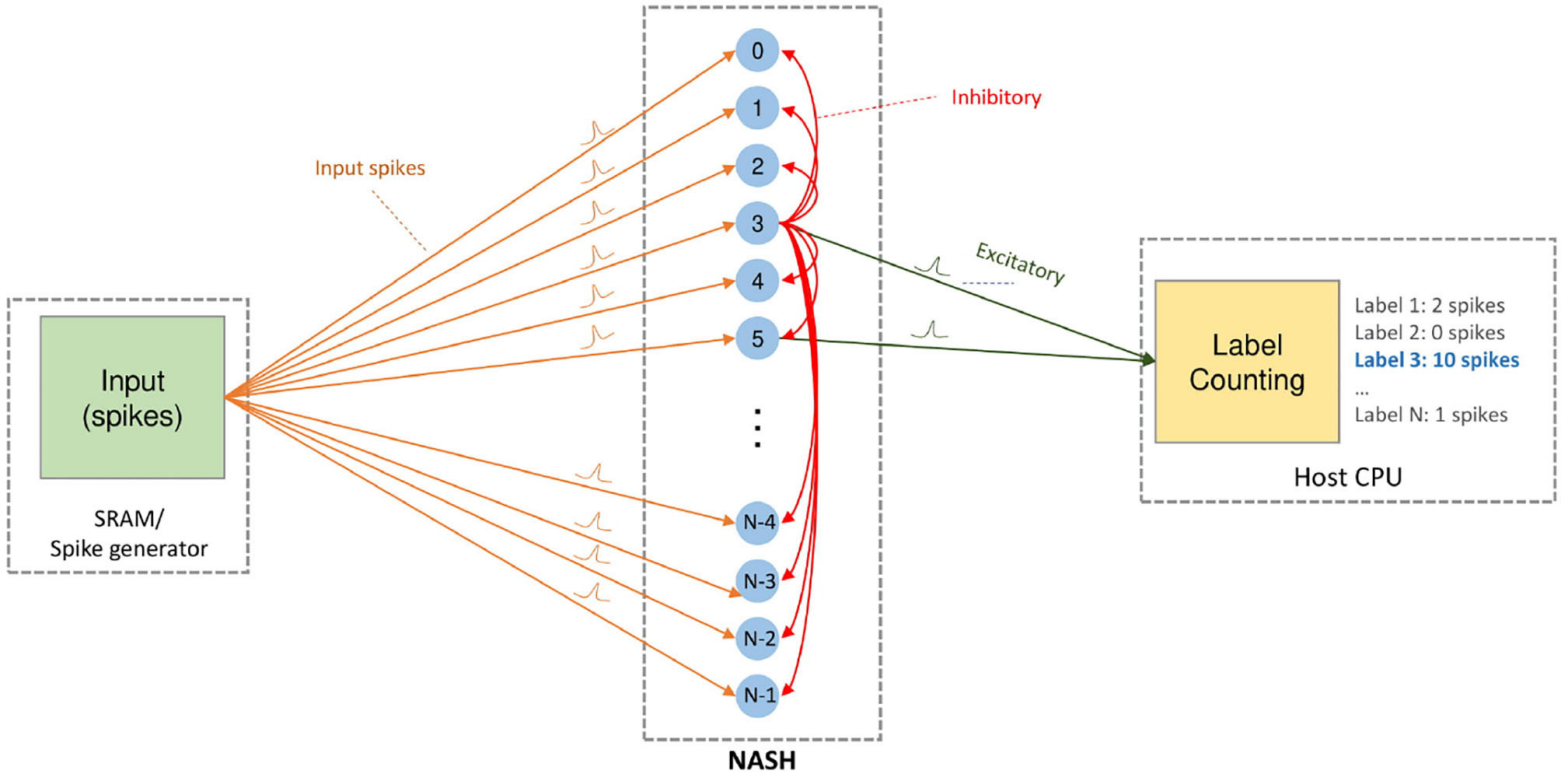
On-chip STDP learning model.

Since the offline learning can be performed with different approaches such as conversion from ANN/CNN (Rueckauer et al., 2017; Sengupta et al., 2019), learning directly with SNN (Yin et al., 2017; Wu et al., 2018), or bio-inspired learning (Hazan et al., 2018) (i.e., STDP, SDSP), different approaches can be adopted for our R-NASH system. Despite being able to load pre-trained weights and parameters to perform inference on our R-NASH, online learning is also supported. Here, the online unsupervised STDP with winner-take-all mechanism is adopted (Diehl and Cook, 2015). Once a neuron fires, it goes to the refractory mode, and an inhibitory spike is broadcasted to others to reduce the membrane potential. Figure 6 shows our system using STDP learning. The input spikes can be stored in SRAM and loaded to the system or generated by the host CPU. Our 3D-NoC interconnect performs the transmission of spikes. Once a neuron fires, it sends the spike to the host CPU to be counted. At the end, the label with the maximum number of spikes become the selected label.

**Figure 7:**
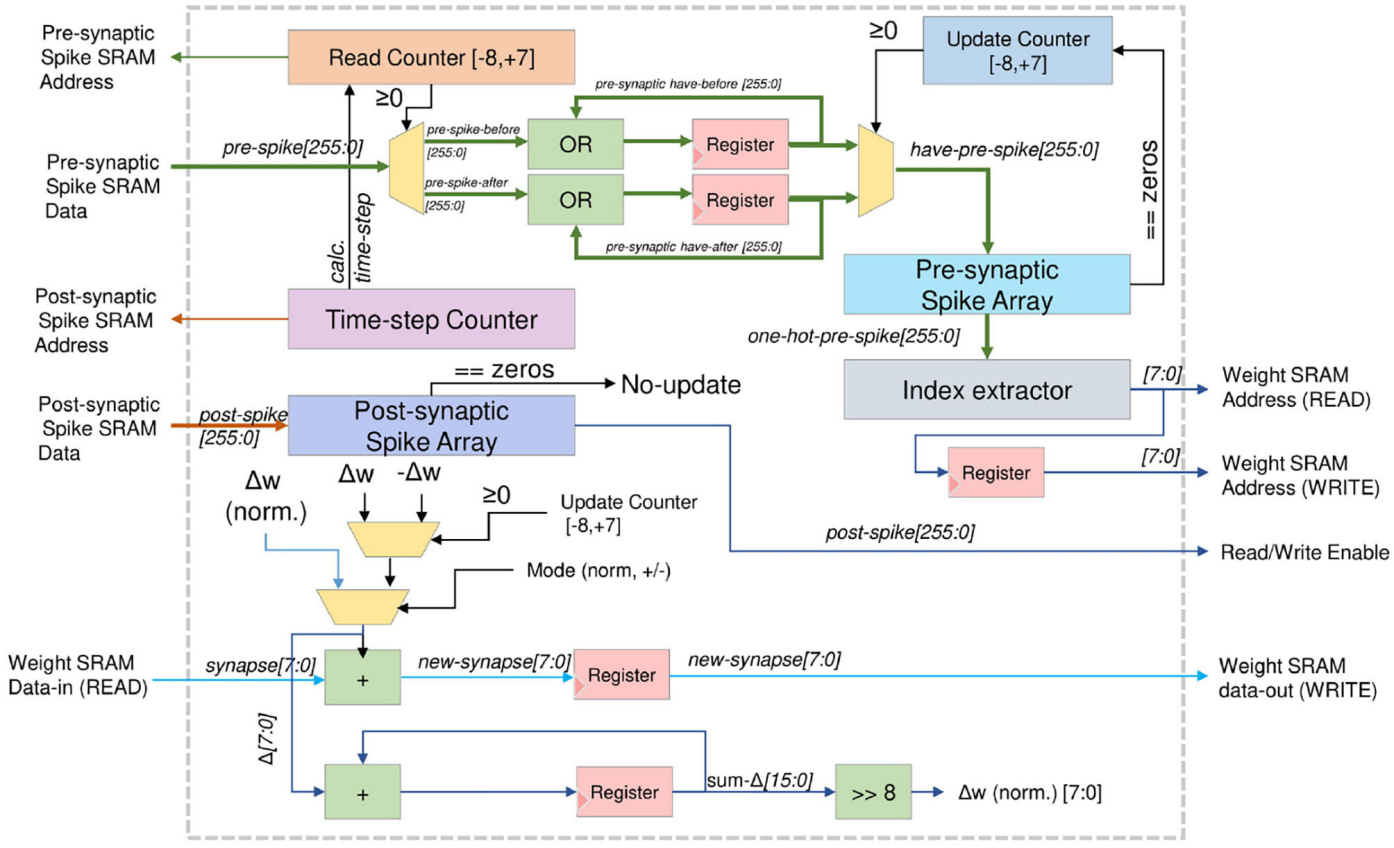
On-chip STDP learning architecture.

Figure 7 shows the online STDP learning block. To reduce complexity, we only adopt a simple STDP mode where the weight of synapses are adjusted to a fixed value based on the presynaptic spike's relative arrival time. If the presynaptic spike from neuron *i* arrives before the event of a postsynaptic spike of neuron *j*, the synapse weight between neuron *i* and *j* (*w*_*ij*_) is increased by a fixed Δ*w* value. On the other hand, if the event of the presynaptic spike from neuron *i* arrives after the event of a postsynaptic spike of neuron *j*, the weight is reduced by a fixed Δ*w* value.

R-NASH implements various reconfiguration using two methods: *(1) adaptive threshold:* once the neuron fires, its threshold is increased by a specific range, but decays if the neuron doesn't fire, *(2) weight normalization:* the average weights of a neuron are unchanged during the learning period. However, due to hardware architecture limitations, R-NASH cannot deliver a high resolution like the floating-point computation unit of CPU or GPU. For adaptive threshold, we use an additional adder in the LIF neuron to adjust the threshold. The adjustment value (Δ*Thres*.) in Figure 5 is selected based on whether the neuron fires or not. As the neuron fires, the threshold is increased until it reaches its maximum value. Otherwise, it decays until it reach its minimum value. By using an adaptive threshold, the neuron's firing pattern can balance with the incoming rates. Howbeit, unbalanced weights can lead to a neuron with higher weight values having a maximum firing rate, which consequently inhibits other neurons from firing. This makes the system fail to learn in a winner-take-all mechanism.

**Figure 8:**
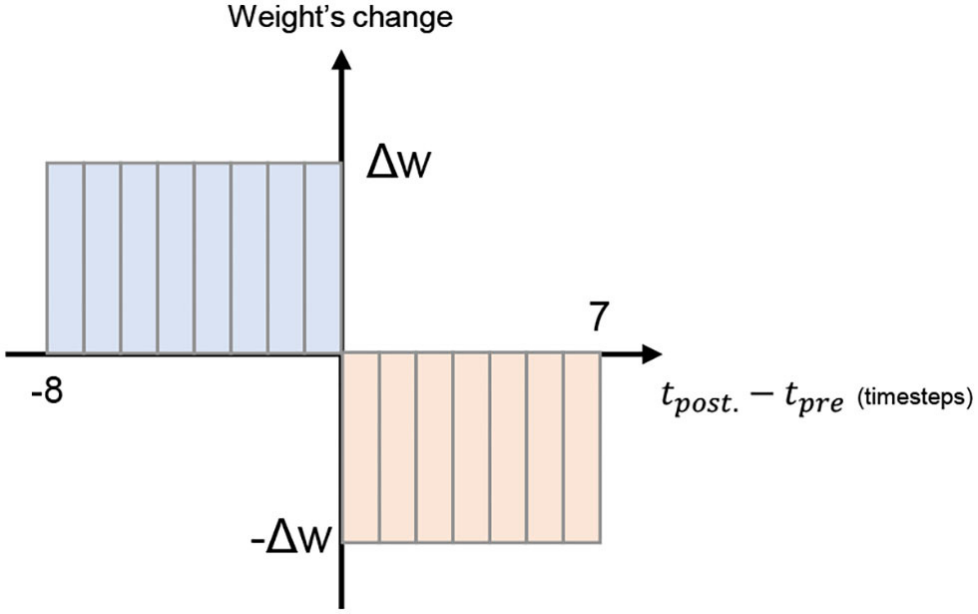
On-chip STDP learning mechanism.

The updating mechanism of the STDP learning is shown in Figure 8. Here, we illustrate with 16 timesteps and fixed weight change to reduce the overall complexity.

### 3.3. Genetic Algorithm for Neurons Mapping on R-NASH Hardware

This section presents the mapping method of the R-NASH system. As we break the neuromorphic system into groups of neurons connected via a Network-on-Chip, dividing and placing are essential issues since they can heavily affect the overall system performance. For instance, placing two connected neurons far apart can lead to a critical delay path in the system. Consequently, the system needs to wait for the spike to travel a long distance before forwarding it. This also increases the power consumption and introduces more thermal dissipation in the packet-switching network. When addressing the issues of mapping, several design factors, including computation, communication, and memory, must be carefully considered. Since the computation on-chip is embedded in nodes with this architecture, we can quickly realize that communication is the most critical problem. If the data is not fed fast enough to the parallel computation unit, the system encounters a communication bottle-neck.

Since the multi-core system's mapping issue is NP-hard (non-deterministic polynomial-time hard), we propose an optimization method using a simple Genetic Algorithm (GA) because ILP is NP-complete and the heuristic search is factorial. At the beginning, the algorithm randomizes K mapping solutions. After having K mapping solutions, it enters G generations of improvement. In each generation, the GA performs several steps as shown in Algorithm 1. During the G generations, the algorithm first removes the incorrect mappings (i.e., requires more computing units than the designed node or has not mapped all computations). The GA algorithm computes the cost function as the following communication cost:

(3) $$\begin{array}{r} F_{cost}=\overset{W}{\underset{i=0,j=0}{\sum }}d_{ij}\times c_{ij} \end{array}$$

where *d*_*ij*_ and *c*_*ij*_ are the distance and the connection status between neuron *i* and *j*. Since the data transfer is in a multi-cast manner at each node, *c*_*ij*_ is the connection between two PEs. B's best communication cost are then selected out of K mapping solutions. We can consider the communication cost as the reverse of the fitness function.

**Algorithm 1:**
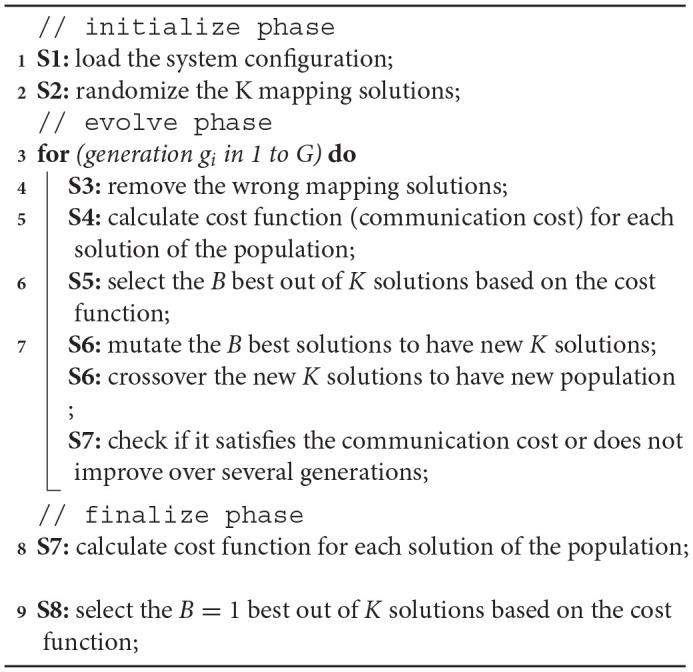
Proposed Genetic Algorithm for Neurons Mapping.

**Figure 9:**
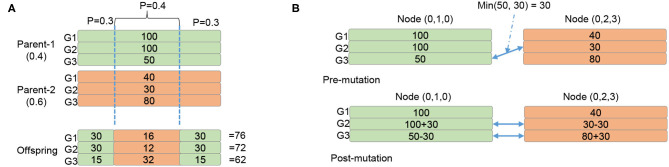
Crossover and mutation method for GA mapping. **(A)** Crossover. **(B)** Mutation.

After having B best solutions, they are crossed over to obtain K solutions. Here, we keep the original B solutions and create a K-B solution using the crossover. The crossover method is shown in Figure 9A. Assuming the crossover probability is 0.4 (randomly picked), the offspring takes 0.4 of a parent and 0.6 of another parent to generate its mapping. Here, the neurons are clusterized in groups (i.e., layers in multiple-layer networks) that could share the pre-synaptic and post-synaptic neurons. Assuming there are three groups in the system and Figure 9A shows two parents' mapping with the configurations for three groups [G1, G2, G3] as [100, 100, 50] and [40, 30, 80]. After the crossover process, an offspring is generated as 60% of [100, 100, 50] plus 40% of [40, 30, 80] which is [76, 72, 62].

After the crossover generates K solutions, the GA mutates the K solutions to generate mutated configurations (under a certain probability). Figure 9B shows how we mutate a configuration. Since the number of neurons mapped in each layer and in each PE is constant, we must maintain it. In Figure 9B we describe a case where the G3 of the node (0, 1, 0) is randomly mutated. To maintain the number of neurons mapped, we randomize the G2 of the node (0, 2, 3) for processing. We find that the minimum of two configurations is 30, which means we can reduce both configurations by 30. Meanwhile, by reducing G3 of (0, 1, 0) by 30, we increase G2 by 30, and G3 of (0, 2, 3) by 30. Note that the reduction value can be randomized between 0 and 30. However, our experiment works best with the minimum one.

After mutation, we re-update the configuration to match the number of unused neurons. Then, we check whether we satisfy the communication cost in the specification. If the communication cost is good enough, we can end the mapping. The GA is also completed after G generations.

### 3.4. Run-Time Maintenance

As previously discussed, robustness is the primary target of R-NASH neuromorphic hardware. Therefore, R-NASH also provides a comprehensive set of fault-tolerance features. In general, there are three significant parts of the R-NASH system that need to be protected: (1) data integrity, (2) interconnect, and (3) computing engine. First, we discuss how R-NASH protects against data corruption. Then we describe the fault tolerance feature for the communication in R-NASH.

#### 3.4.1. Reliability Issue of Large Scale Neuromorphic System

Naturally, spiking neural networks can deal with single or several defective neurons during their operations. However, it will be a critical issue if a considerable number of neurons failed. For instance, defected neurons that consistently fire will affect the overall accuracy of the system.

Assuming the failure probability of a single neuron is $P_{f}^{n}$. In other words, the healthy probability of a neuron is $1-P_{f}^{n}$. Assuming the system has *N* working neurons, its probability of healthy is equal to the probability of having all *N* neuron healthy:

(4) $$\begin{array}{r} P_{h}^{system}={\left(1-P_{f}^{n}\right)}^{N} \end{array}$$

**Figure 10:**
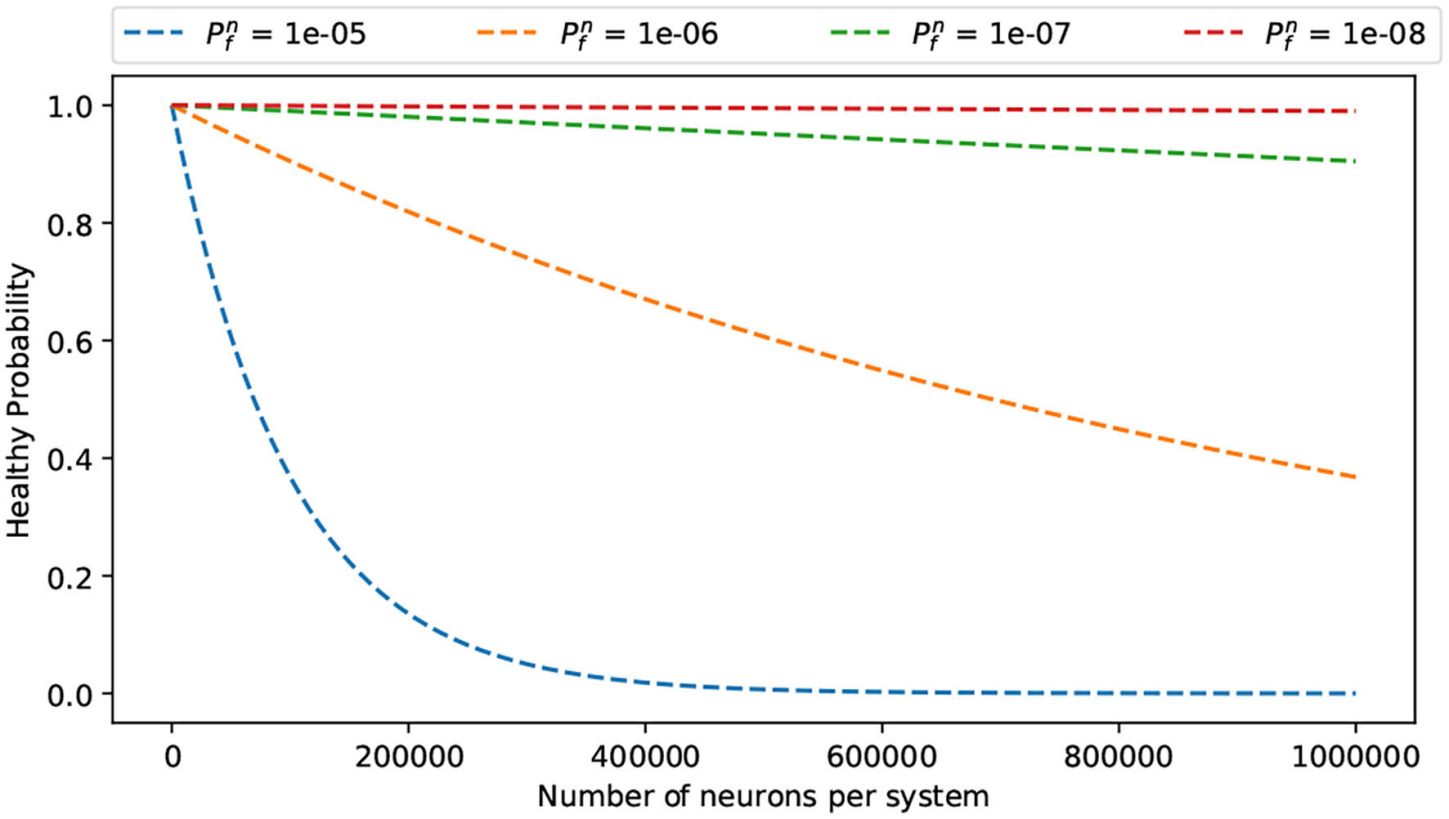
Reliability of large scale system.

Since we target a neuromorphic system with a large number of neurons (i.e., *N* could be thousands or millions), the system's reliability becomes a challenging goal. Figure 10 illustrates the overall system healthy probability under different neuron failure probability and system scale. As we can observe, even with a low failure probability as 10^−6^, when scaling to one million neurons, the system's survival rate is around 0.4. In summary, scaling up the system leads to accumulated failure rates that introduce the reliability issue.

Besides the accumulated failure rate, a large-scale system and 3D-ICs also encounter thermal issues as the operating temperature could be higher than conventional small-scale and 2D-IC systems. In Dang et al. (2021), the normalized fault rate analyses show by increasing the operating temperature from 70°C to 90°C; the fault rate is doubled. By accelerating the failure rates ($P_{f}^{n}$ is increased), the system reliability can be lower.

Moreover, as non-volatile memory (NVM) technologies are considered an emerging technology for neuromorphic systems, it also introduces the reliability issue as NVM has low lifetime reliability than conventional SRAM. In Zhao et al. (2020), the authors present the reliability issue of the resistive crossbar for neuromorphic systems.

#### 3.4.2. Data Integrity Protection

One of the most basic protections in any highly reliable system is to be resilient against data corruption. Here, we classify the data into two types: (1) one-time load data and (2) transferring data. While one-time load data such as weights and thresholds are essential, their accuracy can be maintained by storing them in on-chip SRAM or registers. Here, an error correction code can be used to protect these types of data. However, from our investigation we discovered that soft errors have little impact on the overall accuracy. Therefore, these types of data can be left unprotected and can periodically be written to ensure correctness. The other type of data is the one that is transferred among the system. In particular, spikes and synchronizations are the significant types of flits. The initialization of the system is also essential. Synchronization flits are critical since they ensure the correctness of the system. To protect this type of data, the interconnect supports two sets of SECDED (single error correction, double error detection) (22, 16) (Hsiao, 1970). As a result, in transferring 32-bit of data, the 3D-NoC needs to transfer 44 bits, as this allows our R-NASH to be resilient against one fault per set, and to be alert against two faults per set. In other words, our 3D-NoC allows 2-bit correction and 4-bit detection at its best. By protecting the data, we can ensure the system works with a good level of confidence.

#### 3.4.3. Communication Protection

As we mentioned in the previous section, the data is protected in our R-NASH system, thanks to two sets of SECDED(22,16) (Hsiao, 1970). Moreover, R-NASH also protects the communication infrastructure with the following features. First, by protecting the defective buffer, a technique named Random Access Buffer (Ahmed and Abdallah, 2014) is used to isolate a faulty buffer from the read and write process. Furthermore, if the crossbar is defective, there is a backup link in the crossbar to allow communication (Ahmed and Abdallah, 2014). If the input port, output port, or the whole router is defective, a fault-tolerant routing algorithm can recover the system (Ahmed and Abdallah, 2014).

#### 3.4.4. Fault-Tolerant Neurons Mapping Scheme

**Figure 11:**
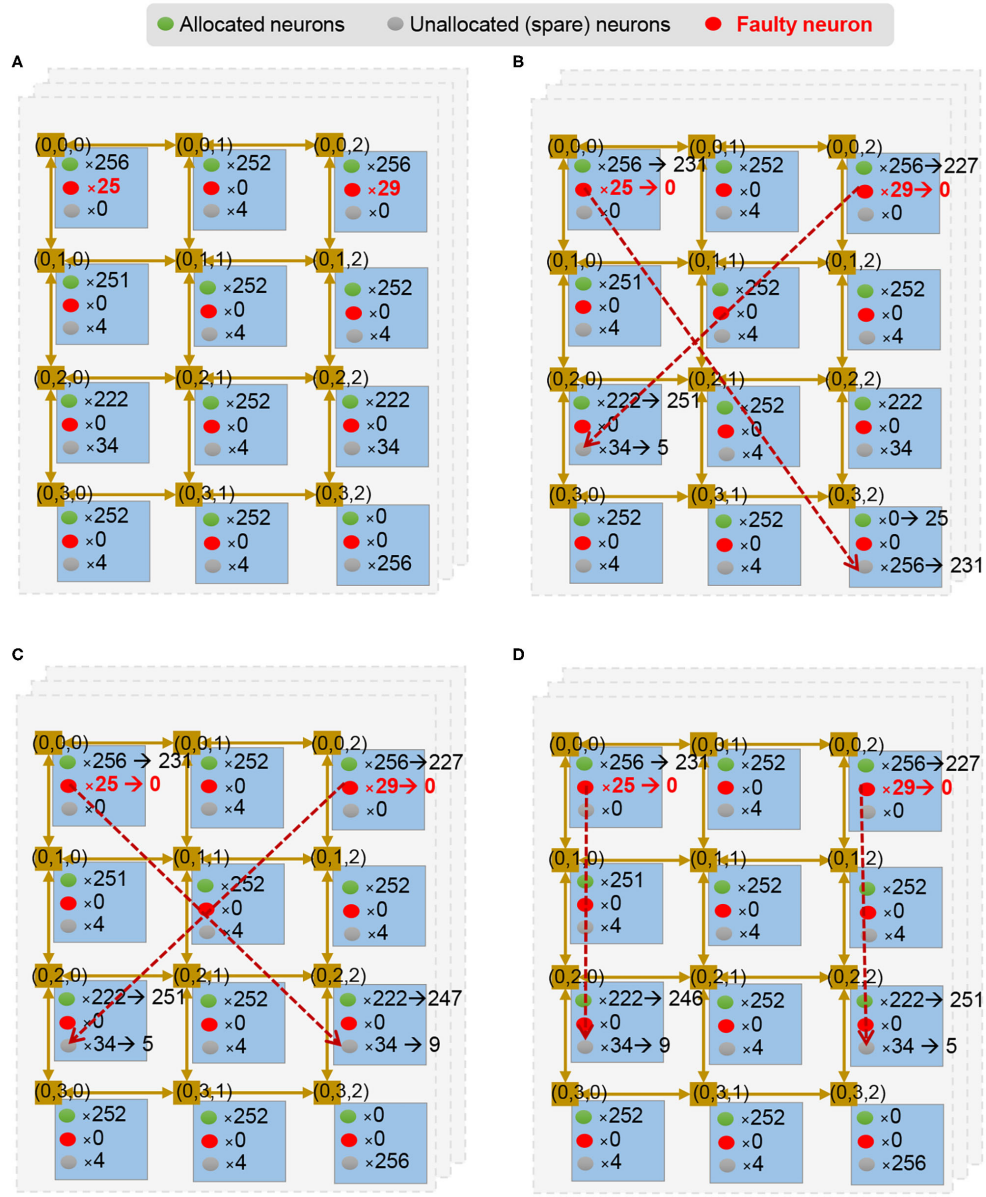
Illustration of fault-tolerance remapping of the Genetic Algorithm. **(A)** Unmapped and free neurons per node. **(B)** A randomized mapping solution. **(C)** Mutating by finding a shorter distance for a flow; **(D)** Mutate by swapping destination of a flow.

Another issue in large-scale neuromorphic architecture is the fact that a given module can develop uncorrectable faults during runtime. This means that the module is corrupted and cannot be used to obtain even graceful accuracy degradation. Therefore, we present a method to remap the neuromorphic system under such faulty circumstances. To protect the faulty neural computing unit against defects, we use two strategies: (1) a node of neuron has some spare neurons (and their weight SRAM), and (2) there is a spare node in the system. Once a neuron/node fails, R-NASH can remap the neuron/node to the spare one and keep its operation. Figure 11A shows an example of a layer in this configuration. Here, each node has a different number of spare neurons. Once there are failed ones, R-NASH maps them to spare neurons. Figure 11B shows the 25 faulty neurons of node (0, 0, 0) remapped into node (0, 3, 2). The number of spare neurons in a node (0, 3, 2) is reduced from 256 to 231 neurons. Since the mapping method already exists, we can use the usual SNN mapping method for replacing neurons. However, the remapping of SNN can provide an alternative approach, since there are new factors in faulty situations. For instance, we might want to reduce the downtime (repairing time) or limit memory transfer within the system. On the other hand, disconnected regions due to faulty network sections can be problematic for mapping. Due to these reasons, we also consider the conventional method: *(1) Greedy Search:* all faulty node run once to find the replacement; *(2) Max-flow min-cut:* convert the problem to the multi-source multi-sink problem; and *(3) Genetic Algorithm* by adjusting the existing algorithm for mapping to obtain a more suitable solution. In the scope of this paper, we present the adaptation of the Genetic Algorithm for remapping. Note that three approaches are also implemented and compared in the evaluation section. Besides the communication cost in Equation (3), we introduce the migration cost to reduce the repairing time of the system as follows:

(5) $$\begin{array}{r} M_{cost}=\overset{W}{\underset{i=0,j=0}{\sum }}d_{ij}\times m_{ij} \end{array}$$

where *m*_*ij*_ is the number of migrating neurons between node *i* and *j*. The main reason to adopt the migration cost is to reduce the repair time due to the fact that reloading neuron weights are expensive and can affect the real-timeliness of R-NASH. The max-flow min-cut adaptation actually provides the optimal flow solution, which means the lowest migration cost in the system.

Algorithm 2 shows the proposed Remapping Genetic Algorithm. It consists of three phases: (1) initialize, (2) evolve, and (3) finalize. The initialize phase starts with the first step **S1** where the number of unmapped and free neurons are counted and sent from each node of the system. Figure 11A illustrates an example of a layer after the initial phase. Based on these values, the second step **S2** generates K mapping solutions randomly (i.e., Figure 11B). This step randomizes a node with free neurons and a node with unmapped neurons from the values in **S1**. At the end of step *S2*, the algorithm generates *K* legal mapping solutions. They are not optimal solutions and need to be optimized.

**Algorithm 2:**
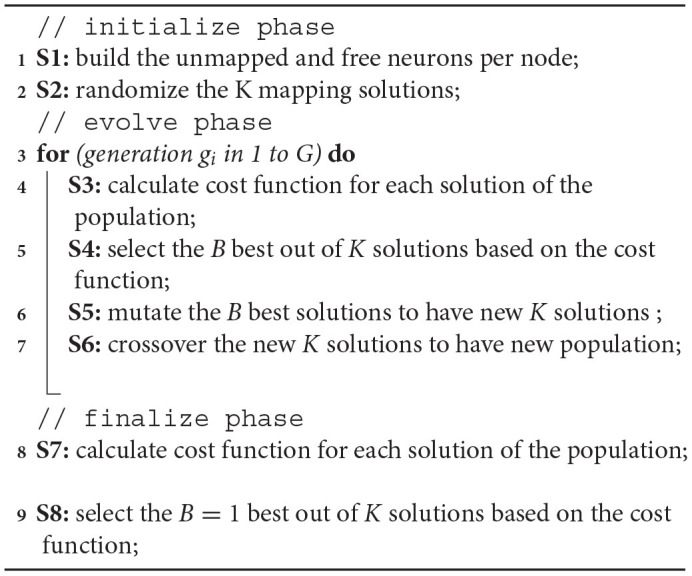
Proposed Genetic Algorithm for remapping SNN.

In the *evolve* phase, the GA method iterates for *G* generations where each generation repeats four steps. At first, step **S3** compute the cost function for each solution. Here, we can adopt only *M*_*cost*_ from Eq. 5. The communication cost *F*_*cost*_ is also calculated for the selection step *S*4. In *S*4, the best *B* solutions in *K* are ranked, and if they have similar *M*_*cost*_ values, their *F*_*cost*_ are considered. Doing so preserves the simplicity of a single objective optimization for GA, while still considering migration and communication costs.

After getting *B* best solutions, it goes through two steps: **S5**-crossover and **S6**-mutation. The crossover step **S5** is performed by mixing two random mapping solutions. It takes 50% of each parent to generate offspring. By doing so, the offspring can inherit the mappings of its two parents.

There are two types of mutations in the mutation step **S6**. First, it finds an immediate random node between two random nodes having a mapping flow. Here, we ensure the immediate node having free neurons is closer to the source node than the destination of the flow. For instance, Figure 11B shows the case where the source node (0, 0, 0) has 25 unmapped neurons and all are mapped to (0, 3, 2) — the destination node. Then, it finds the immediate node (0, 2, 2) with two conditions: (1) there are free neurons in the immediate node (0, 2, 2) and (2) the distance from the source node (0, 0, 0) to the immediate node (0, 2, 2) is smaller than the original flow [(0, 0, 0) to (0, 3, 2)]. The neurons are then remapped to the immediate node instead of the destination. The result can be seen in Figure 11C.

The second mutation is to swap the mapping to have a closer migrating distance (smaller *M*_*cost*_). If there are two flows that can have smaller migrating distance by swapping the destination, the algorithm performs the swap. For instance, Figure 11C shows unmapped neurons in node (0, 0, 0) being mapped to (0, 2, 2), and unmapped neurons in node (0, 2, 0) being mapped to (0, 0, 2). Here, the migrating distances are four for both flows. However, by switching the destination, we obtain a migrating distance of two for both flows, as shown in Figure 11D. After G generations, the algorithm finalizes by selecting only the best solution (step **S7** and **S8**). This solution is used to perform the mapping method. Since the GA might take a long time to complete, we can also allow early termination of the mapping and use the best-found solution. In summary, this GA methodology provides an extension for the optimization problem of remapping faulty neurons. While the mapping algorithm only focuses on the communication cost, GA allows designers to take the migration cost function for the optimization.

## 4. R-NASH Evaluation Results

In this section, we present the performance evaluation of the proposed system. First, the initial mapping issue is addressed to show the efficiency of the GA (genetic algorithm) model. Here, we map multiple layer feed-forward networks to different 3D-NoC sizes from 4 × 4 × 4 to 10 × 10 × 10. To understand the effect of having different node sizes, we map the same system into different node sizes (256, 128, 64, and 32 neurons per node) and topologies. We also investigate the difference between 3D and 2D topologies to illustrate the benefits of 3D structure. To improve the robustness of the neuromorphic hardware, the proposed fault-tolerant mapping is presented and compared with conventional works like greedy search and max-flow min-cut. Third, we present the hardware complexity for our system with NANGATE 45nm and FreePDK45 TSV library. We then present both offline and online training for the MNIST dataset in our R-NASH neuromorphic hardware. For offline learning, the offline trained weights of a multiple-layer feed-forward neural network were converted and used for classification in R-NASH. The task of online learning was performed using the on-chip STDP with winner-take-all mechanism, thanks to the inhibitory connections. Finally, we discuss the pros and cons of our approach.

### 4.1. Initial Mapping Evaluation

In this section, we show our initial mapping evaluation. The input configuration is fed to the platform, which helps generate the 3D-NoC mapping. A host PC later uses the output configuration to configure the R-NASH.

#### 4.1.1. Mapping Over Different 3D-NoC Sizes

To understand the GA method's efficiency for initial mapping, we first compare it with three linear mapping solutions. We adopt the linear mapping method from SpiNNaker (Jin, 2010) and implement it for the 3D topology to get Linear X, Linear XY, and Linear XYZ, which represent the priority direction of the linear mapping. The mapping configuration can be found in Table 1. Note that there are fixed spare neurons (20% per node) and an extra node (the highest index node) that are not used for mapping and can be used to tolerate faults later in section 4.2.

**Table 1:** Configuration for the mapping evaluation^1^.

**Parameter**	**Value**
# neurons per node (E)	256
# nodes (N)	4 × 4 × 4 to 10 × 10 × 10
# spare neurons (R)	0.2 × X
# spare node	1
# faults (k)	0.05×X, 0.10×X, 0.15×X, and 0.20×X
SNN # layers	4
SNN configuration^1^	784:0.5^*^(W-10): 0.5^*^(W-10): 10

^0^ *X: number of neurons in R-NASH*.

1 *MLP model. For example, the SNN configuration for E=64 and 4 × 4 × 4 is 784:1633:1633:10*.

**Figure 12:**
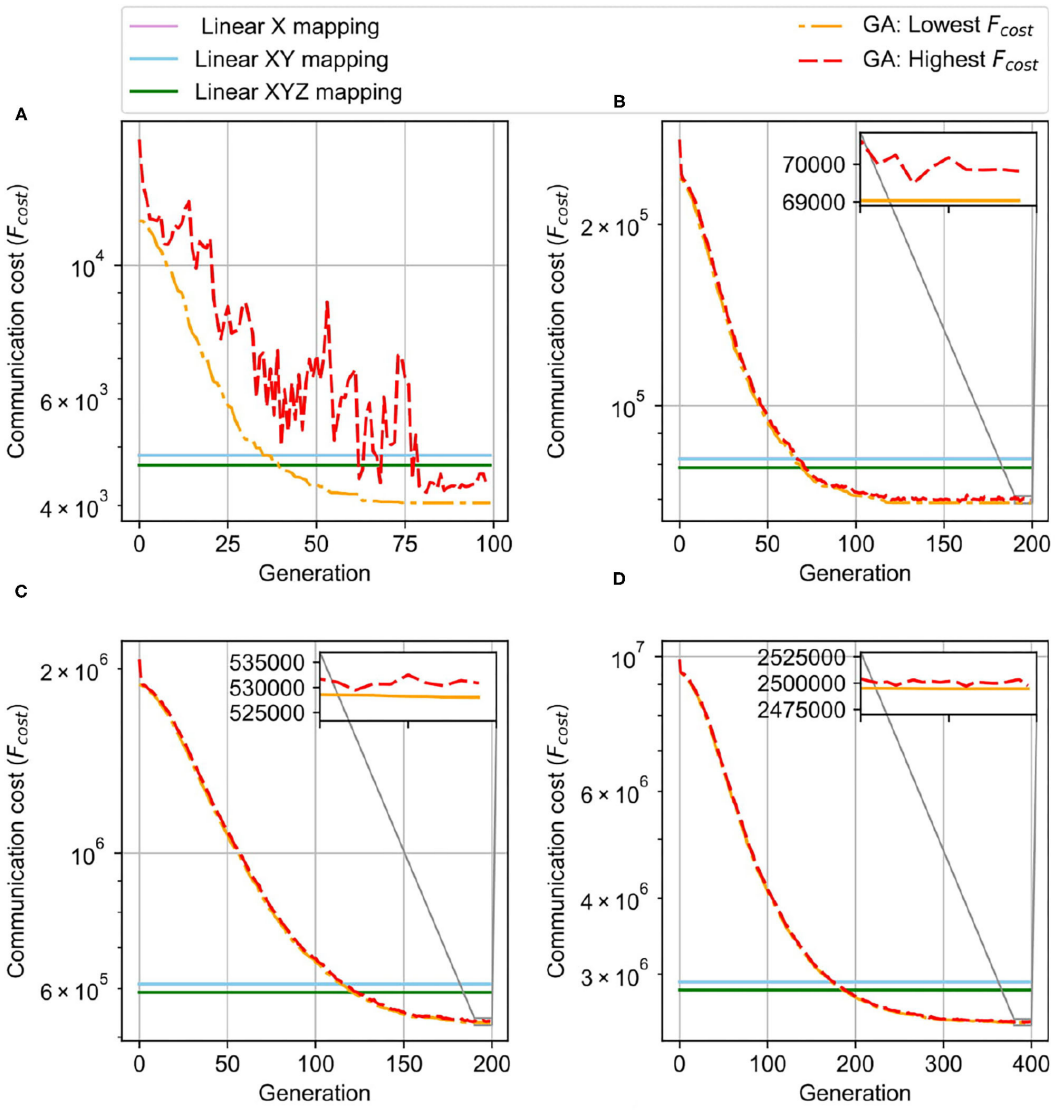
Genetic Algorithm Result for initial mapping. **(A)** 4 × 4 × 4 NoC-based, 256 neurons/node. **(B)** 6 × 6 × 6 NoC-based, 256 neurons/node. **(C)** 8 × 8 × 8 NoC-based, 256 neurons/node. **(D)** 10 × 10 × 10 NoC-based, 256 neurons/node.

Here, we run GA with population K=100, the best B=5, and the mutation rate of 0.5. Figure 12 shows the result of GA in comparison with the linear mapping methods. With a small network size of 4 × 4 × 4, after nearly 60 generations, the GA saturates at a point, and the lowest communication cost stays unchanged over the rest of the generations. The final communication cost is lower than both manual mapping solutions, and the overall cost is 0.4× the manual mapping. With larger NoCs, it is easy to understand that it needs more generations to be lower than the linear mapping. While 4 × 4 × 4 takes around 60 generation to converge, 6 × 6 × 6, 8 × 8 × 8, and 10 × 10 × 10 need around 120, 180, and 320 generations, respectively, to be stable. We can observe that linear mapping methods have significantly higher communication costs in all tested cases than the genetic algorithm ones.

#### 4.1.2. Mapping Over Different Node Sizes

**Figure 13:**
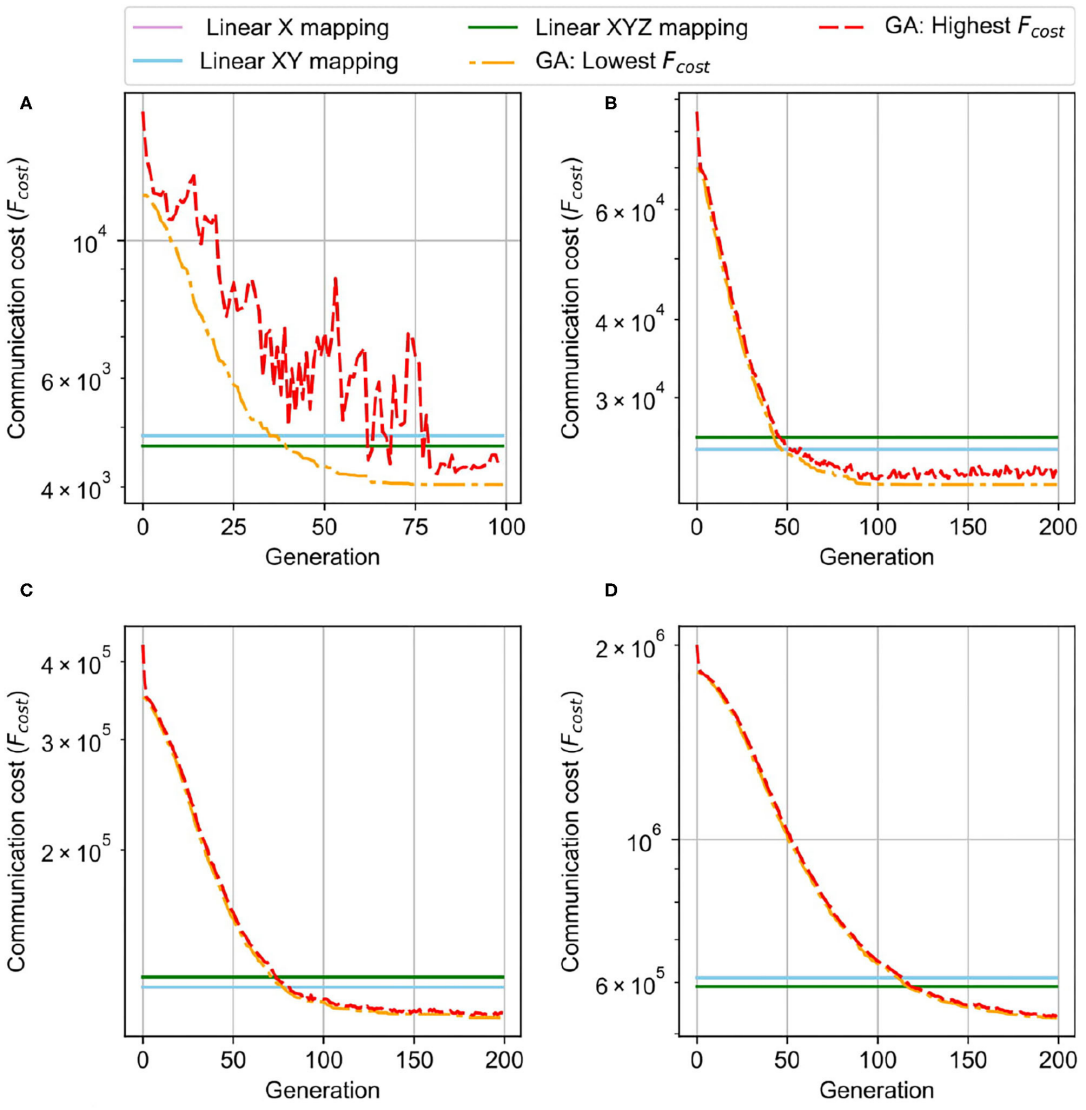
Genetic Algorithm Result of the initial mapping of 3D NoC-based. **(A)** 4 × 4 × 4, 256 neurons/node. **(B)** 4 × 4 × 8, 128 neurons/node. **(C)** 4 × 8 × 8, 64 neurons/node. **(D)** 8 × 8 × 8, 32 neurons/node.

Figure 13 illustrates the mapping results over different node sizes from 32 to 256. To maintain the same system size (the same number of neurons), we vary the 3D-NoC size from 4 × 4 × 4 to 8 × 8 × 8. As we can observe in Figure 13, the smaller NoC benefits the smaller distances between nodes, which can reduce the communication cost. The smallest network size provides the lowest communication cost (4039). By increasing the network's size and reducing the node's size, the communication cost keeps increasing. With 128, 64, and 32 neurons per node, the communication costs are 21,768, 107,838, and 529,440, respectively. We do not need to send multiple unicast flits for spikes by placing neurons in the same layer into a node. Instead, sending a single flit and distributing it to all nodes can significantly reduce the traffic. However, we would like to note that scaling up the number of neurons per node is not unlimited due to the limitation on crossbars and bottleneck on on-chip communication. Moreover, having a large size node also leads to the following main disadvantages: (1) lower operating frequency due to a long critical path; (2) difficulty to place and route due to complex structure and macro SRAM and (3) long distances between nodes could also reduce the performance of the NoC. Typically, the neuromorphic cluster varies between 256 and 1,024 neurons per node.

#### 4.1.3. Comparison Between 3D and 2D in Initial Mapping

**Figure 14:**
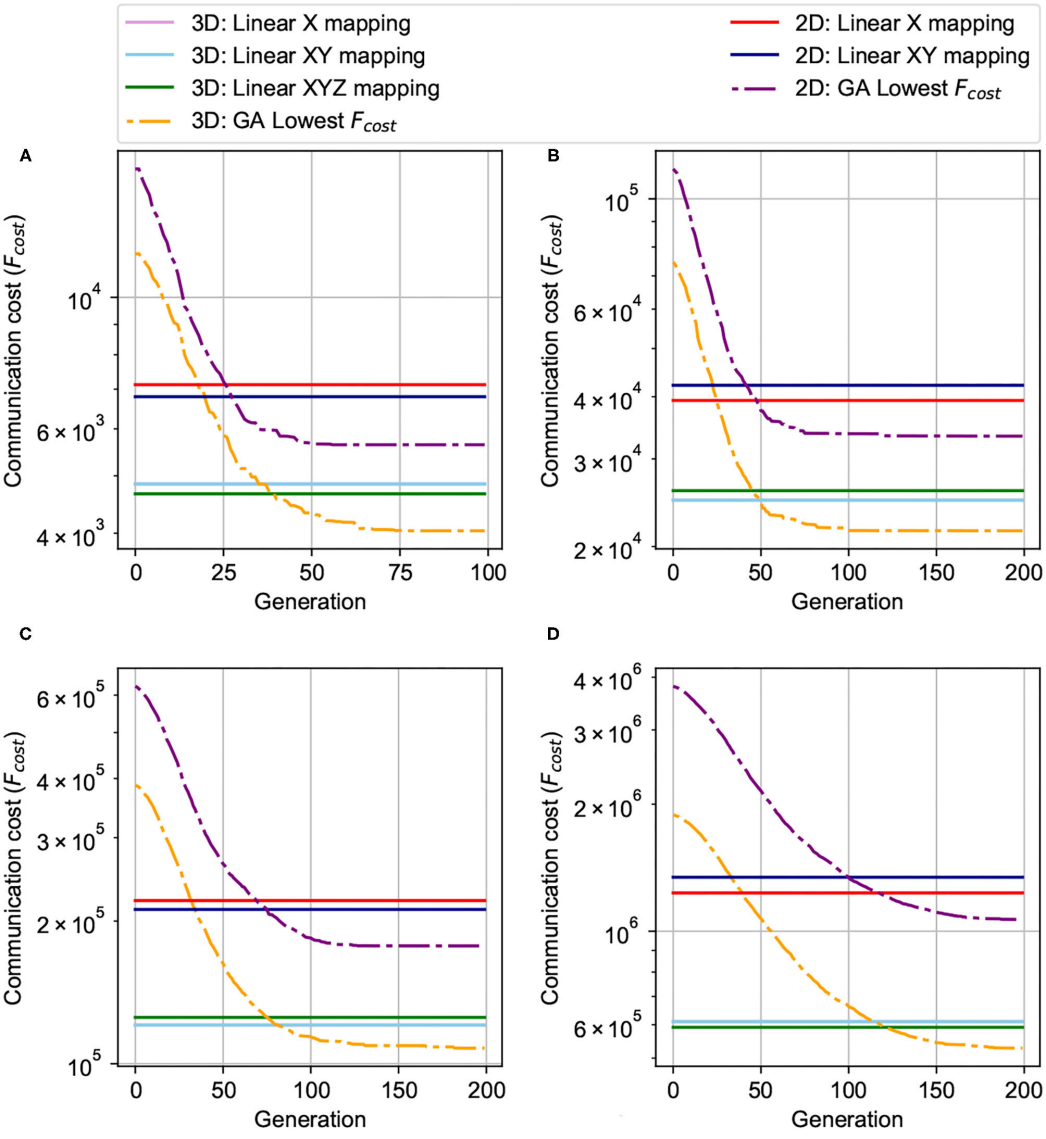
Comparison between 3D and 2D mapping. **(A)** 64 nodes (4 × 4 × 4 and 8 × 8) NoC-based, 256 neurons/node. **(B)** 128 nodes (4 × 4 × 8 and 8 × 16) NoC-based, 256 neurons/node. **(C)** 256 nodes (4 × 8 × 8 and 16 × 16) NoC-based, 256 neurons/node. **(D)** 512 nodes (8 × 8 × 8 and 16 × 32) NoC-based, 256 neurons/node.

We compare the communication cost between 3D-NoC-based nerormorphic (3D-R-NASH) and 2D-NoC- based neuromorphic (2D-R-NASH) systems under the same linear mapping and GA in Figure 14. We keep the same node size as 256 neurons per node for a fair comparison and the change between the NoC sizes. We compare 3D and 2D networks with the same number of nodes (64, 128, 256, and 512).

As can be observed in Figure 14, mapping on a 3D structure leads to a significantly smaller communication cost. In all test cases (64, 128, 256, and 512 nodes), the performance of GA on 3D is 1.4-2.0× smaller than the 2D ones. Even with linear mappings (X, XY, or XYZ), 3D still dominates the 2D. This is due to the nature of 3D bringing much shorter traversal paths between regions of the chip. Spikes can travel much faster on 3D, and this could be translated into better performance and lower power in 3D.

### 4.2. Fault-Tolerant Mapping

In this section, the *Genetic Algorithm* for remapping is evaluated and compared with 1-hop and N-hop *Greedy Search* (GS) and MFMC: *max-flow min-cut* adaption to understand its efficiency. The fault-tolerant mapping is written in Python as it reuses the configuration from the initial mapping phase. Fault information from R-NASH is an input for this fault-tolerant mapping. The *Greedy Search* runs each node once and looks for a spare node within one (1) hop range or in the entire system (N-hop) with the shortest distance. On the other hand, the MFMC adaption solves the maximum flow from faulty neurons to spare neurons; therefore, MFMC provides the most optimal solution in terms of migration cost. The configuration of the evaluation is shown in Table 1. In this evaluation, we measure three major parameters: *(1) mapping rate:* the ability to map the faulty neurons to the spare ones; *(2) average spike transmission cost (F*_*cost*_*)*: the average distance of all connections and *(3) Migration cost* *M*_*cost*_*: the amount of read/write neurons needed to adapt the system*.

**Figure 15:**
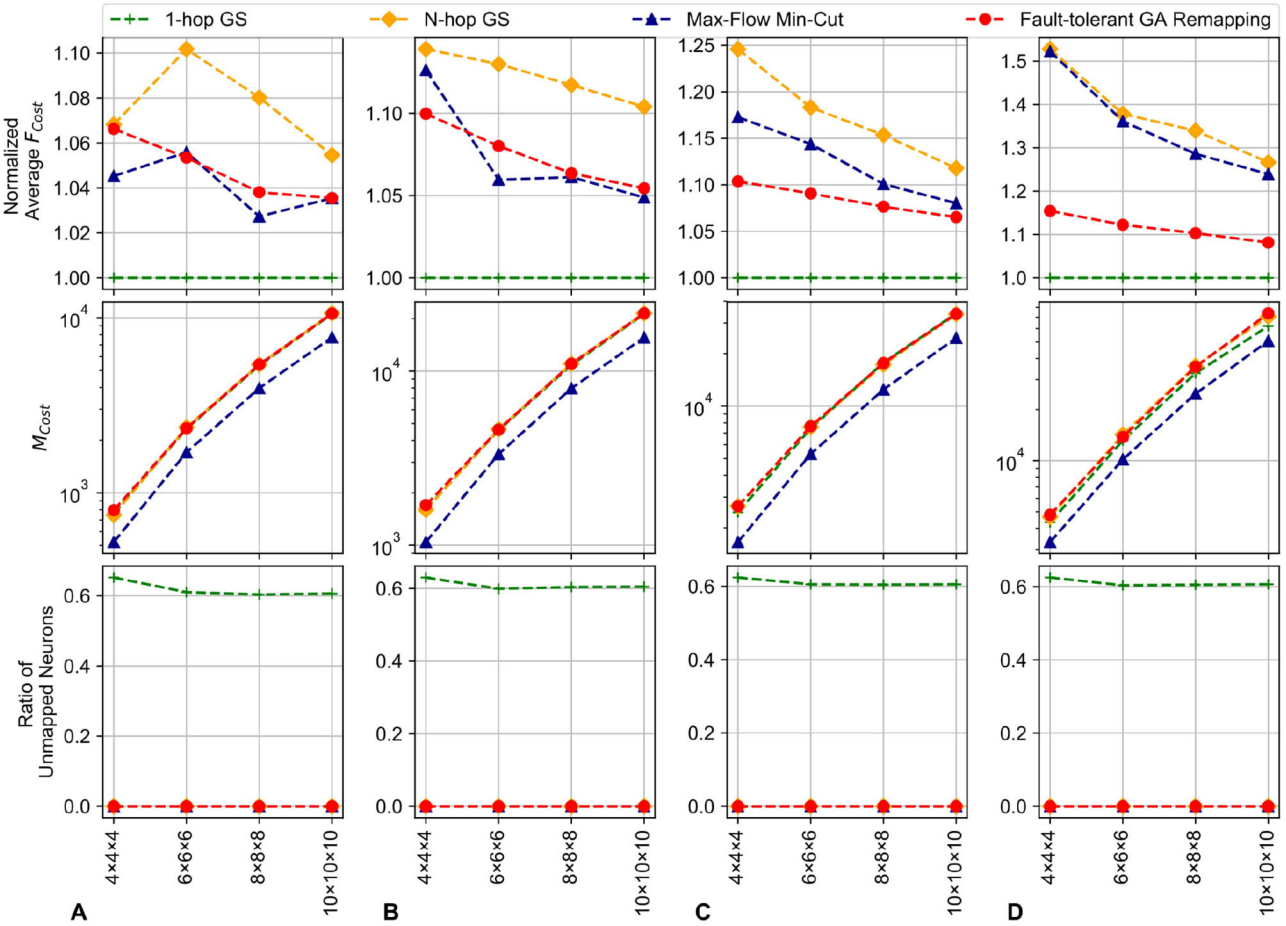
Output mapping for the migrated neurons with random fault patterns in 3D-NoCs. The system has 256 neurons per node; 20% of the neurons are spares with 1 redundant node without any allocated neuron at 0% fault rate. **(A)** 5% defect rate. **(B)** 10% defect rate. **(C)** 15% defect rate. **(D)** 20% defect rate.

Figure 15 illustrates the results for the proposed system for 3D-NoC configurations (see Table 1). As shown in Figure 15, our GA method can map all faulty neurons to the spare ones regardless of the size or topology. We have to note that the MFMC algorithm is not optimal for communication costs and 1-hop *Greedy Search* can only map around 60% (around 80% with the worst cases) of the faulty neurons. This is because 1-hop *Greedy Search* only runs once in each node and looks for one of its neighbor to map. Meanwhile, the N-hop *Greedy Search* and Genetic Algorithm can map all neurons. The average *F*_*cost*_ (communication cost) also varies between different approaches. Since the 1-hop GS mostly fail to map the neurons, the average communication distance per neuron is unchanged. For other methods, the average *F*_*cost*_ fluctuates between different sizes. However, as we can observe in Figure 15, they are reduced when we increase the size of the NoC. This is because when we increase the size of the NoC, the impact of moving neurons is reduced. The effects are also smaller, with smaller fault rates (k values). We can even notice the communication cost is maintained with remapping. However, a slight reduction can be observed with the migration-based algorithm. Also, GA seems to have a better average *F*_*cost*_ since it optimizes *F*_*cost*_ as the second factor. In conclusion, we have shown the efficiency of adapting the GA (genetic algorithm) for solving the remapping problem. Although the proposed GA in some cases still has a larger *F*_*cost*_ than others, it has shown efficiency on both migration cost and communication cost. Moreover, it shows efficiency even with high defect rates where the communication cost is much lower. On the other hand, using the max-flow min-cut to solve the problem is also reasonable for such a situation.

### 4.3. Hardware Complexity

Table 2 shows the hardware complexity of the proposed R-NASH node. The NI which supports the mapping method is integrated with the neuron cluster and the 3D-NoC router. As shown in Table 2, the additional LUTs for AER and Address take up 23.35% and 28.83% of the Network Interface area respectively. The overhead of these two LUTs is relatively small. On the other hand, the NI, which supports migration techniques, only occupies 25.95% of the tile area without the SRAM (neuron cluster + network interface).

**Table 2:** Hardware complexity of the proposed R-NASH node.

**Module**		**Area**	**Max Freq**.
		**(*μm*^2^)**	**(*MHz*)**
	AER LUT	16,747	-
Network Interface	Address LUT	20,768	-
	Total	72,032	699.30
Neuron Cluster		205,608	751.87
		64KB SRAM	-
3D-NoC router (Dang et al., 2020b)		41,739	537.63
Vertical TSVs (up and down)		2,901.1136	-

**Figure 16:**
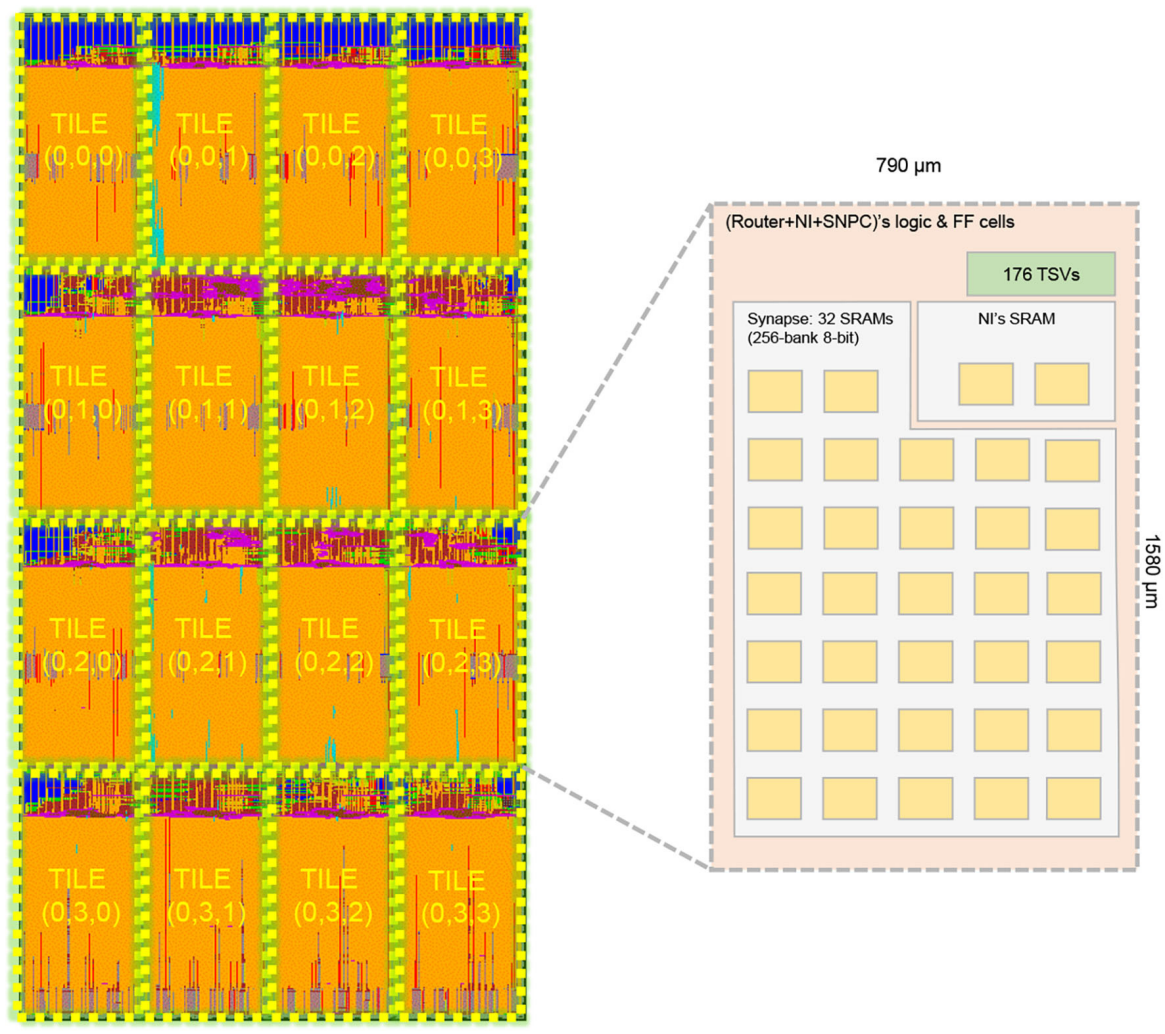
Layout of a 4 × 4 R-NASH layer. A tile's size is 790μ*m* × 1, 580μ*m*.

Figure 16 illustrates our sample layout for a 4 × 4 NoC-based SNN layer with migration support. The cluster's configuration is 256 spike inputs in AER format, 8-bit synapse weight, 32 physical neurons, 32 synapse crossbars for each cluster. Here, each crossbar is implemented with a 256-bank 8-bit dual-port SRAM using OpenRAM. We only integrate 32 neurons per node to have a reasonable Place&Route time and a visual layout. To support 3D-NoC inter-layer interconnect, we use TSV from FreePDK3D45 with the size of 4.06μ*m*^2^ × 4.06μ*m*^2^ and the Keep-out-Zone is 15μ*m*^2^ × 15μ*m*^2^ for each TSV. As can be observed in the layer's layout, 80% of the area is for placing macro SRAM. Since the design of the LIF neuron is light-weight, the most complicated part is the crossbar.

Howbeit, the NI requires two dedicated SRAMs for converting the AER from local value to a global one and a destination lookup table. We can further optimize the design's footprint by reducing the bit-width of a synapse or using an alternative memory approach (eDRAM, STT-RAM, or memristor). Moreover, we add more stacking layers dedicated to memory, which allows us to have a smaller footprint.

### 4.4. System Validation

This section presents the result of online and offline training for our R-NASH system. As the 3D-NoC aims to model a complex neuromorphic system, the conventional MNIST classification neural networks are too small to map. Therefore, in the validation section, we have scaled up the feed-forward neural network's hidden layer to map it into our R-NASH. We first show the offline conversion from a feed-forward ANN to our SNN. The weight and other parameters are loaded into the system using a memory interface. Then, the online STDP method is presented. The initial weights, which are randomized and normalized, are loaded in our R-NASH system.

#### 4.4.1. Offline Feed-Forward Network

For the offline training, we use the feed-forward network 784:1024:1024:10 and 784:1024:1024:1024:10 for the MNIST dataset. We fixed the hidden layer to 1024 to fit the 10-bit SRAM model for the hardware design. Here, we use a 3D-NoC of 4 × 4 × 4 with 64 neurons/node from the output of the initial mapping in Figure 13. Since 2058 and 3,082 neurons are used in the two networks, we reserve the remaining ones as the spare neurons for tolerating potential defects. There is one spare node at (3, 3, 3) and spare neurons of around 31/32 for the first network and 2/3 for the second network in all active nodes. Although scaling the 3D-NoC and the number of neurons per node can support a bigger network-size, we only adopt the above feed-forward size to avoid large SRAM models. Using sparse synapses could reduce the SRAM size; however, we only target to validate R-NASH's operation. The training and inference phases in software are written in MATLAB. The R-NASH is described and simulated in Verilog HDL with the converted weight from the MATLAB models.

**Figure 17:**
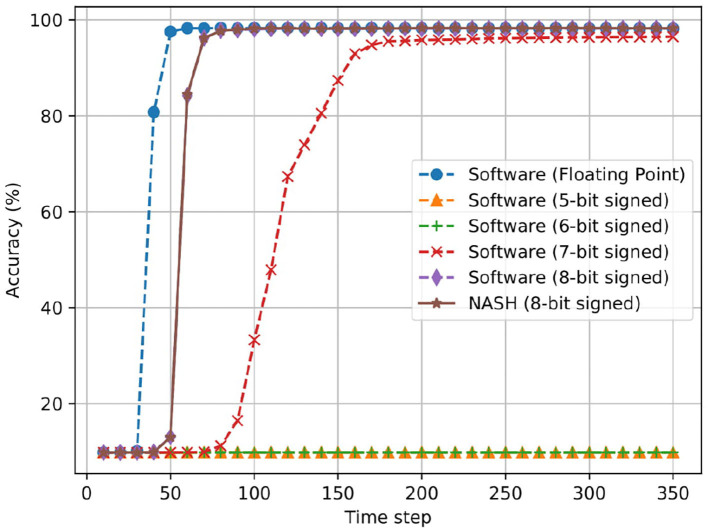
Accuracy result of offline training for MNIST dataset with the network model 784:1024:1024:10.

Figure 17 shows the accuracy results of 784:1024:1024:10 on the R-NASH system in comparison with the software version. The R-NASH system uses an 8-bit signed weight representation which gives similar results to the converted version in Matlab. The total number of time steps is 350 (1 ms per time step in the simulation). Here, we also evaluate the fixed point SNN in software where we clip the least significant bit in representation. We also consider our R-NASH, where 8-bit signed fixed-point values are converted to an integer value to enable hardware implementation. At first, we can easily see the drop in accuracy when comparing the floating-point SNN and the fixed point ones. The reductions are significant when the number of representing bit is <5. The main reason is that the more extensive and deeper network will accumulate the values' differences, which results in more inaccurate results. Nevertheless, we can easily see that an 8-bit signed fixed point is the best for implementation and provides nearly identical accuracy at the end with slower response time.

**Figure 18:**
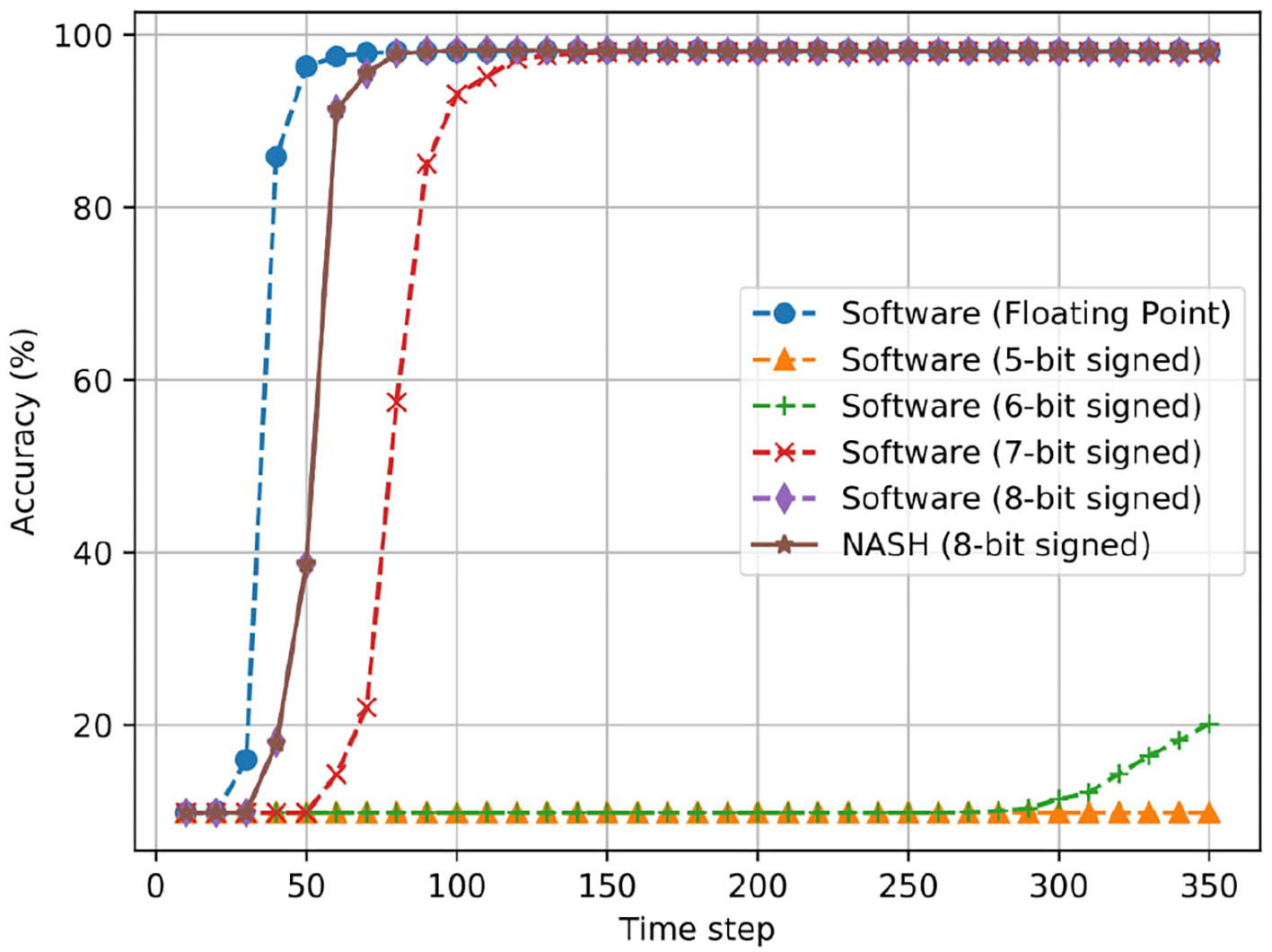
Accuracy result of offline training for MNIST dataset with the network model 784:1024:1024:1024:10.

Figure 18 illustrates the case of three hidden layer network (784:1024:1024:1024:10). Here, we can observe a similar behavior as the first network. The R-NASH system provides a similar result as the software in floating point. The 7-bit fixed point version now can have final result of inference close to the floating point; however, it needs over 100 time steps to converge.

In summary, R-NASH can perform inference of pretrained and converted networks, and the result is identical to the software version. Note that R-NASH saturates around the 55th time step in both cases. If the system cuts the operation at this point, it could save nearly 85% of the computation time. By using the clock gating method (Mahmoodi et al., 2008), the energy could be saved at zero data switching activity.

### 4.5. Unsupervised STDP

In this section, we evaluate the online STDP method for the same MNIST benchmark. Here we adopt the network in Diehl and Cook (2015) with the recurrent version that could be found in the work of Hazan et al. (2018). Furthermore, we simplify the architecture to be identical to the hardware implementation. The network size of Diehl and Cook (2015) is 784:N:N while our network is 784:N. Since the number of neurons is not significant enough to scale to a 3D-NoC, we only use 1 × 4 × 4 3D-NoC and 64 neurons/node. There are two versions with *N* = 100 and *N* = 400, mapped into 2 and 4 nodes. Since there is no sparse connection, the communication cost stays unchanged with any neuron placements. The training and inference in software is written in Python with our customized LIF neurons.

For testing purposes, we adopt the BindsNet (Hazan et al., 2018) platform to build the RTL-like version of the LIF neuron to train and test on our PC. After completing the testing and debugging phase, the Verilog model train is performed and compared with the golden reference software. In the software model of SNN (Diehl and Cook, 2015), the authors used the adaptive weight change (Δ*w* = *w* × learning_rate); however, it is not suitable for our hardware STDP due to two reasons: (1) the resolution of the weight (8-bit) is too small to use the same principle and (2) the architecture for the multiplication is too complicated. Therefore, we use the fixed weight change here. We also evaluate the method for more understanding.

Table 3 shows the accuracy of the software version of STDP learning and our hardware STDP SNN. Comparing the R-NASH model and the software (Diehl and Cook, 2015), we could observe a drop in accuracy by using our RTL model. This is due to the much simpler hardware model and lower resolution (fixed 8-bit for weight, 16-bit for membrane potential, 16-bit for normalizing).

**Table 3:** Accuracy result of STDP learning for SNNs.

**N**	**Floating point software**	**R-NASH**
100	79.44%	71.32%
400	88.87 %	84.05%

**Figure 19:**
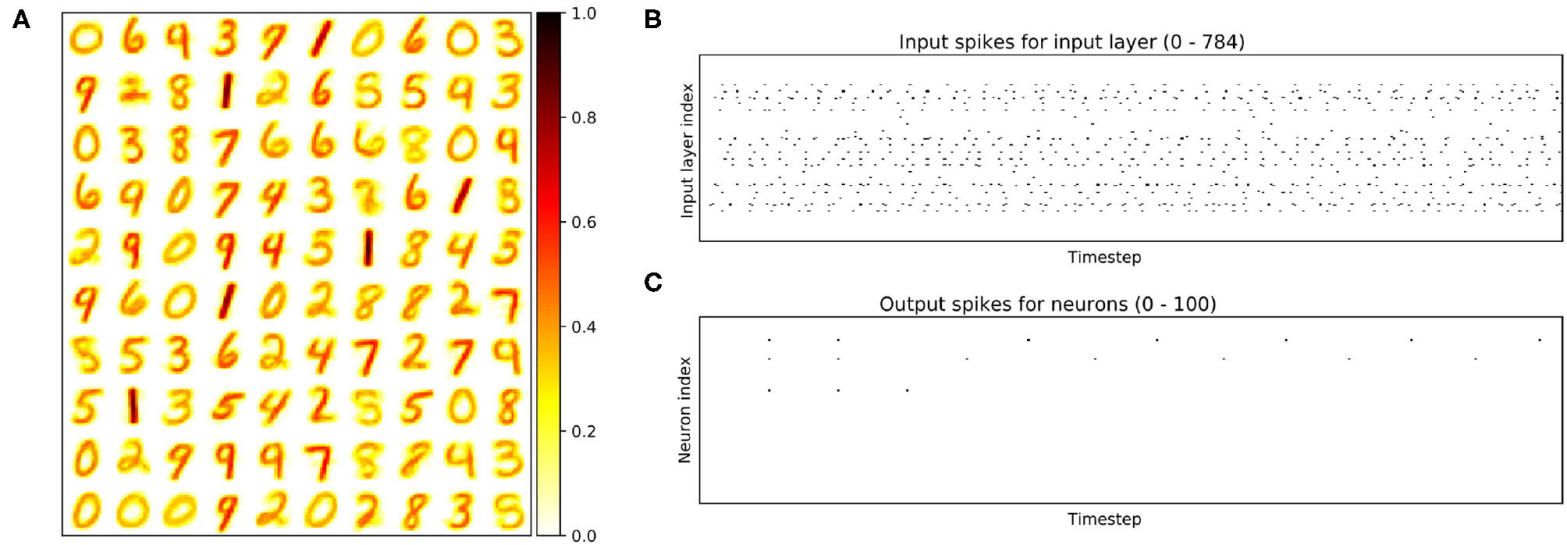
Illustration of the STDP learning model. **(A)** the final weights. **(B)** Illustration of input spikes for the first test image (number 8). **(C)** Illustration of output spikes.

Figure 19 illustrates the weight with *N* = 100 and the input (output) spikes extracted from our R-NASH software model. The weights have been adapted into the MNIST. However, there are some drawbacks due to the hardware model's simplicity. For instance, there is some weight with a similar distribution. As a result, these neurons fire simultaneously and continue to fire during the following time steps. Figure 19 illustrates that three neurons continue to fire during the 350 simulated timesteps. Moreover, the weights keep changing during the training time due to the pure STDP without intervention. We can certainly observe that some weights are mixed of two numbers (i.e., 8 and 5, 1 and 7).

Table 4 depicts the comparison between works on neuromorphic systems with the MNIST dataset. For on-chip learning, our STDP learning accuracy with 100-neurons has lower accuracy in contrast to other approaches. However, by having 400 neurons in the model, its accuracy is compatible with different online learning approaches. On the other hand, the conversion from ANN dominates other learning methods with around 98% accuracy.

**Table 4:** Comparison result of MNIST accuracy on hardware neuromorphic systems.

**Work**	**Kim et al., 2015**	**Frenkel et al., 2018**	**Seo et al., 2011**	**Ours**	
Neuron Model	IF	LIF and Izhikevicz	LIF	LIF	
Learning	Stochastic	On-chip	On-chip	On-chip	ANN
Method	gradient descent	stochastic SDSP	STDP	STDP	conversion
Configuration	4 cores	1 core	1 core	1/2 core	9/13 cores
neurons/core	64	256	256	256	256
Synapse precision	4,5,14 bit	4 bit	1 bit	8-bit	
Accuracy (%)	84	84.5	77.2	79.4/ 84.5	98.2/98.8
Technology	65-nm	28-nm FD-SOI CMOS	45-nm SOI-CMOS	45-nm CMOS	
Energy per SOP	N/A	8.4pJ (0.55V)	N/A	11.3pJ (1.1V)	

### 4.6. Discussion

We have presented the design and the platform of a novel 3D neuromorphic system (R-NASH). Moreover, we demonstrated the scalability of the proposed approach and its ability to tolerate faults during run-time. In this section, we discuss the existing problems and potential solutions:

First, the 3D-ICs are expected to have higher operating temperatures than the 2D-ICs due to the silicon layers' organization. In addition, 3D-ICs suffer from thermal dissipation. Large-scale neuromorphic systems also introduce high-density power consumption, which leads to high temperature. Therefore, optimizing the operating temperature is one of the critical issues. In Arka et al. (2021), the authors presented a MOO approach to optimize the operating temperature for 3D multi-core systems. As an optimization method, the Genetic Algorithm can surely solve multiple objective optimization (MOO) problems (i.e., using NSGA-II Deb et al., 2002), however, as it is a complex issue, further investigation is needed.

Second, the scalability of the address range in R-NASH is currently limited to 3-bit per address. To have a better range, having an extra bit to represent the address is necessary. For instance, by adopting a 64-bit format for 3D NoC flit, the extra 32-bit can be distributed into address, neural mask, or AER fields. In TrueNorth (Akopyan et al., 2015), the flit consists of the offset between the source and the destination address; therefore, there is virtually no limitation on the address range. However, this way of addressing has two drawbacks: (1) the memory access is no longer globally accessible as one node can only reach a specific range, and (2) there are limited options for long-range synapses. By having a limited access range of memories, downloading weight for inference need a different mechanism. Moreover, the fault-tolerance and the initial mapping issues are also under constraints.

The STDP training has not been efficient so far for multiple layer neural networks. In Lee et al. (2018), the authors have presented a method to train the kernel of convolutional neural network with STDP the update mechanism is still complicated for full-hardware implementation. In Shi et al. (2021), a hardware-friendly method has been introduced; however, the procedure is based on layer-based error backpropagation, which is not bio-plausible. Consequently, STDP-based multiple-layer network training is still an open problem for future researches.

In our R-NASH design, a neuron can send its spikes to a high number of downstream neurons. The routing can be multicasting by replicating the flits during operation. In the NI, we also support a relaying protocol that allows the incoming spikes to be relayed to the nearby cores. However, there is a limitation on the number of upstream neurons that can send spikes to a neuron. Since the synaptic SRAM size is unchanged after manufacture, the number of memory cells to store the weight is limited. Consequently, it not possible to extend the size of up-stream neurons.

Tree topology has been considered as more efficient than mesh topology (Merolla et al., 2013) as the packet relayed peak at *O*(*n*^3/2^) for unicast mesh and *O*(*n*) for the multicast tree. On average, mesh unicast is still *O*(*n*^3/2^) while tree multicast drop to *O*(*n*). Consequently, lesser packets are being relayed in tree topology thanks to its natural structure. However, while shifting to 3D-ICs, mesh topology can naturally extend into the third dimension by simply adding the vertical connection (up and down). Meanwhile, tree-topology in Merolla et al. (2013) support left, right, and top directions, which cannot naturally extend into the third dimension. Also, as mesh topology can provide escape channels if there is faulty ports or faulty neuron, tree-topology is extremely sensitive with fault as a single fault can cut a part of the tree out of communication.

The problem of large fan-in and fan-out is an important one to be addressed. Bamford et al. (2010) presented a method tailored for the address-event receiver that allows a large axonal fan-out structure for neuromorphic systems. The technique can help solve the sizeable fan-in issue that our neuromorphic system is limited on the SRAM size. Meanwhile, Zamarreño-Ramos et al. (2012) shows a multicasting mesh for AER that allows communication in neuromorphic systems via 2D-Mesh topology. Compared to our approach, this work provides destination-driven routing, which is similar to our routing but in 2D Mesh. They also offer source-driven routing as a multicasting approach. This method requires a look-up to help the router understand the branch structure of the routing path. The source-driven routing can be efficient; however, it requires a large memory block in each router for routing, which is problematic for large-scale systems. Moreover, as we focus on the fault-tolerant routing in this work, table-based routing has two significant drawbacks. First, it introduces extra faulty elements. The faulty routing tables change the routing path, which may create a deadlock or livelock scenario. Second, any change in neuron location leads to an extensive amount of table update in the system.

## 5. Conclusion

In this work, we proposed and evaluated a reliable three-dimensional digital neuromorphic system geared explicitly toward the 3D-ICs biological brain's three-dimensional structure toward the design of a cross-paradigm system. Spike timing patterns represent the information in the network, and learning is based on the local spike-timing-dependent plasticity rule. The proposed platform enables high integration density and slight spike delay of spiking networks and features a scalable design. R-NASH is a design based on the Through-Silicon-Via technology, facilitating spiking neural network implementation on clustered neurons based on Network-on-Chip. We provide a memory interface with the host CPU, allowing for online training and inference of spiking neural networks. Moreover, R-NASH supports faults recovery using our fault-tolerant mapping method by optimizing communication and migration costs. We presented the functionality of the system by performing the MNIST dataset classification. Moreover, the R-NASH platform is also presented with the mapping method and fault-tolerance features. The mapping method shows that it can easily outperform manual mapping. On the other hand, we also proposed a genetic algorithm for fault recovery in SNN. Although the proposed work is expected to move neuromorphic computing toward a real-world scenario on large-scale systems, further optimization, such as bit-width reduction, low-power optimization, is needed.

Future works for NASH would focus on real chip fabrication and deployment in a real-world application scenario, such as hand gesture recognition, prosthetic, and robotic arm control. Multiple-objective optimization for several costs such as communication, operating temperature is also considered in our future works. In STDP learning, keeping the weight dynamic is also an essential issue in our future work to balance the area cost and neuron/weight complexity.

## Data Availability Statement

The raw data supporting the conclusions of this article will be made available by the authors, without undue reservation.

## Author Contributions

AB initiated the research, produced the conceptual design and framework. KD conceived and design the principles of the R-NASH. KD wrote the R-NASH code and the initial version of the manuscript. AB and KD contributed to the revisions and produced the final manuscript. Both authors contributed to the article and approved the submitted version.

## Conflict of Interest

The authors declare that the research was conducted in the absence of any commercial or financial relationships that could be construed as a potential conflict of interest.

## References

[B1] Ahmed A. B. Abdallah A. B. (2014). Graceful deadlock-free fault-tolerant routing algorithm for 3D Network-on-Chip architectures. J. Parallel Distribut. Comput. 74, 2229–2240. 10.1016/j.jpdc.2014.01.002

[B2] Akopyan F. Sawada J. Cassidy A. Alvarez-Icaza R. Arthur J. Merolla P.. (2015). TrueNorth: design and tool flow of a 65 mW 1 million neuron programmable neurosynaptic chip. IEEE Trans. Comput. Aided Design Integr. Circ. Syst. 34, 1537–1557. 10.1109/TCAD.2015.2474396

[B3] Arka A. I. Joardar B. K. Kim R. G. Kim D. H. Doppa J. R. Pande P. P. (2021). Hem3d: heterogeneous manycore architecture based on monolithic 3D vertical integration. ACM Trans. Des. Autom. Electron. Syst. 26, 1–21. 10.1145/3424239

[B4] Bamford S. A. Murray A. F. Willshaw D. J. (2010). Large developing receptive fields using a distributed and locally reprogrammable address–event receiver. IEEE Trans. Neural Netw. 21, 286–304. 10.1109/TNN.2009.2036912 20071258

[B5] Banerjee K. Souri S. J. Kapur P. Saraswat K. C. (2001). 3-D ICs: A novel chip design for improving deep-submicrometer interconnect performance and systems-on-chip integration. Proc. IEEE 89, 602–633. 10.1109/5.929647

[B6] Ben Ahmed A. Ben Abdallah A. (2013). Architecture and design of high-throughput, low-latency, and fault-tolerant routing algorithm for 3D-network-on-chip (3D-NoC). J. Supercomput. 66, 1507–1532. 10.1007/s11227-013-0940-9

[B7] Benjamin B. V Gao P. McQuinn E. Choudhary S. Chandrasekaran A. R. Buss J.-M. (2014). Neurogrid: a mixed-analog-digital multichip system for large-scale neural simulations. Proc. IEEE 102, 699–716. 10.1109/JPROC.2014.2313565

[B8] Chen G. K. Kumar R. Ekin Sumbul H. Knag P. C. Krishnamurthy R. K. (2018). A 4096-neuron 1M-synapse 3.8-pJ/SOP spiking neural network with on-chip STDP learning and sparse weights in 10-nm finFET CMOS. IEEE J. Solid State Circ. 54, 992–1002. 10.1109/JSSC.2018.2884901

[B9] Dang K. N Ahmed A. B Abdallah A. B Tran X. (2020a). TSV-OCT: a scalable online multiple-TSV defects localization for real-time 3-D-IC systems. IEEE Trans. Very Large Scale Integ. Syst. 28, 672–685. 10.1109/TVLSI.2019.2948878

[B10] Dang K. N Ahmed A. B Okuyama Y Abdallah A. B. (2020b). Scalable design methodology and online algorithm for TSV-cluster defects recovery in highly reliable 3D-NoC systems. IEEE Trans. Emerg. Top. Comput. 8, 577–590. 10.1109/TETC.2017.2762407

[B11] Dang K. N Ben Abdallah A. (2019). An efficient software-hardware design framework for spiking neural network systems, in 2019 International Conference on Internet of Things, Embedded Systems and Communications (IINTEC) (Gammarth), 155–162.

[B12] Dang K. N. Ahmed A. B. Abdallah A. B. Tran X.-T. (2021). Hotcluster: a thermal-aware defect recovery method for through-silicon-vias toward reliable 3-d ics systems. IEEE Trans. Comput. Aided Design Integr. Circ. Syst. 1. 10.1109/TCAD.2021.3069370

[B13] Davies M. Srinivasa N. Lin T.-H. Chinya G. Cao Y. Choday S. H.. (2018). Loihi: a neuromorphic manycore processor with on-chip learning. IEEE Micro 38, 82–99. 10.1109/MM.2018.112130359

[B14] Deb K. Pratap A. Agarwal S. Meyarivan T. (2002). A fast and elitist multiobjective genetic algorithm: Nsga-ii. IEEE Trans. Evolut. Comput. 6, 182–197. 10.1109/4235.996017

[B15] Diehl P. U Neil D. Binas J. Cook M. Liu S.-C. Pfeiffer M. (2015). Fast-classifying, high-accuracy spiking deep networks through weight and threshold balancing, in 2015 International Joint Conference on Neural Networks (IJCNN) (Killarney), 1–8.

[B16] Diehl P. U. Cook M. (2015). Unsupervised learning of digit recognition using spike-timing-dependent plasticity. Front. Comput. Neurosci. 9:99. 10.3389/fncom.2015.00099 26941637 PMC4522567

[B17] Eliasmith C. Stewart T. C. Choo X. Bekolay T. DeWolf T. Tang Y.. (2012). A large-scale model of the functioning brain. Science 338, 1202–1205. 10.1126/science.1225266 23197532

[B18] Frenkel C. Lefebvre M. Legat J.-D. Bol D. (2018). A 0.086-mm^2^ 12.7-pJ/SOP 64k-synapse 256-neuron online-learning digital spiking neuromorphic processor in 28-nm CMOS. IEEE Trans. Biomed. Circ. Syst. 13, 145–158. 10.1109/TBCAS.2018.2880425 30418919

[B19] Frenkel C. Legat J. Bol D. (2019). MorphIC: a 65-nm 738k-Synapse/mm^2^ quad-core binary-weight digital neuromorphic processor with stochastic spike-driven online learning. IEEE Tran. Biomed. Circ. Syst. 13, 999–1010. 10.1109/TBCAS.2019.2928793 31329562

[B20] Furber S. B Galluppi F. Temple S. Plana L. A. (2014). The SpiNNaker project. Proc. IEEE 102, 652–665. 10.1109/JPROC.2014.2304638

[B21] Furber S. (2016). Large-scale neuromorphic computing systems. J. Neural Eng. 13, 051001. 10.1088/1741-2560/13/5/051001 27529195

[B22] Goldwyn H. J. S Imennov N. Famulare M. Shea-Brown E. (2011). Stochastic differential equation models for ion channel noise in Hodgkin-Huxley neurons. Phys. Rev. E 83, 4190–4208. 10.1103/PhysRevE.83.041908 21599202 PMC3279159

[B23] Hazan H. Saunders D. J. Khan H. Patel D. Sanghavi D. T. Siegelmann H. T.. (2018). BindsNET: a machine learning-oriented spiking neural networks library in Python. Front. Neuroinforma. 12:89. 10.1147/rd.144.0395 30631269 PMC6315182

[B24] Hsiao M.-Y. (1970). A class of optimal minimum odd-weight-column SEC-DED codes. IBM J. Res. Dev. 14, 395–401.

[B25] Ikechukwu M. O. Dang K. N. Abdallah A. B. (2021). On the design of a fault-tolerant scalable three dimensional noc-based digital neuromorphic system with on-chip learning. IEEE Access. 9, 64331–64345. 10.1109/ACCESS.2021.3071089

[B26] Jin X. (2010). Parallel Simulation of Neural Networks on Spinnaker Universal Neuromorphic Hardware. The University of Manchester.

[B27] Joseph J. M. Samajdar A. Zhu L. Leupers R. Lim S.-K. Pionteck T.. (2021). Architecture, dataflow and physical design implications of 3D-ICs for DNN-accelerators, in International Symposium on Quality Electronic Design (ISQED) (Santa Clara, CA), 1–7.

[B28] Kim J. K. Knag P. Chen T. Zhang Z. (2015). A 640m pixel/s 3.65 mw sparse event-driven neuromorphic object recognition processor with on-chip learning, in 2015 Symposium on VLSI Circuits (VLSI Circuits) (Kyoto: IEEE), C50–C51.

[B29] Lee C. Srinivasan G. Panda P. Roy K. (2018). Deep spiking convolutional neural network trained with unsupervised spike-timing-dependent plasticity. IEEE Trans. Cogn. Dev. Syst. 11, 384–394. 10.1109/TCDS.2018.2833071

[B30] Lee H. G. Chang N. Ogras U. Y. Marculescu R. (2008). On-chip communication architecture exploration: a quantitative evaluation of point-to-point, bus, and network-on-chip approaches. ACM Trans. Design Autom. Electr. Syst. 12, 1–20. 10.1145/1255456.1255460

[B31] Levin J. A. Rangan V. MALONE E. C. (2014). Efficient Hardware Implementation of Spiking Networks. Patent No. US 2014/0351190 A1, Filed May 1, 2014, Pub. Date Nov. 27, 2014.

[B32] Mahmoodi H. Tirumalashetty V. Cooke M. Roy K. (2008). Ultra low-power clocking scheme using energy recovery and clock gating. IEEE Trans. Very Large Scale Integr. Syst. 17, 33–44. 10.1109/TVLSI.2008.2008453

[B33] Merolla P. Arthur J. Alvarez R. Bussat J.-M. Boahen K. (2013). A multicast tree router for multichip neuromorphic systems. IEEE Trans. Circ. Syst. I Regular Papers 61, 820–833. 10.1109/TCSI.2013.2284184

[B34] Ogbodo M Vu T Dang K Ben Abdallah A. (2020). Light-weight spiking neuron processing core for large-scale 3D-NoC based spiking neural network processing systems, in 2020 IEEE International Conference on Big Data and Smart Computing (BigComp) (Busan), 133–139.

[B35] Panth S. A. Samadi K. Du Y. Lim S. K. (2014). Design and CAD methodologies for low power gate-level monolithic 3D ICs, in Proceedings of the 2014 International Symposium on Low Power Electronics and Design (La Jolla), CA, 171–176.

[B36] Purves D. Augustine G. Fitzpatrick D. Hall W. LaMantia A.-S. McNamara J. (2008). Neuroscience. Sunderland, MA: Sinauer Associates.

[B37] Rueckauer B Lungu I. -A. Hu Y. Pfeiffer M. Liu S. -C. (2017). Conversion of continuous-valued deep networks to efficient event-driven networks for image classification. Front. Neurosci. 11:682. 10.3389/fnins.2017.00682 29375284 PMC5770641

[B38] Schemmel J. Brüderle D. Grübl A. Hock M. Meier K. Millner S.. (2010). A wafer-scale neuromorphic hardware system for large-scale neural modeling, in Proceedings of 2010 IEEE International Symposium on Circuits and Systems (Paris), 1947–1950.

[B39] Scholze S. Schiefer S. Partzsch J. Hartmann S. Mayr C. Höppner S.. (2011). Vlsi implementation of a 2.8 gevent/s packet-based aer interface with routing and event sorting functionality. Front. Neurosci. 5:117. 10.3389/fnins.2011.00117 22016720 PMC3191349

[B40] Sengupta A. Ye Y. Wang R. Liu C. Roy K. (2019). Going deeper in spiking neural networks: vgg and residual architectures. Front. Neurosci. 13:95. 10.3389/fnins.2019.00095 30899212 PMC6416793

[B41] Seo J Brezzo B. Liu Y. Parker B. D. Esser S. K. Montoye R. K.. (2011). A 45 nm CMOS neuromorphic chip with a scalable architecture for learning in networks of spiking neurons, in 2011 IEEE Custom Integrated Circuits Conference (CICC) (San Jose, CA), 1–4.

[B42] Shi C. Wang T. He J. Zhang J. Liu L. Wu N. (2021). Deeptempo: a hardware-friendly direct feedback alignment multi-layer tempotron learning rule for deep spiking neural networks. IEEE Trans. Circ. Syst. II Exp. Briefs 68, 1581–1585. 10.1109/TCSII.2021.3063784

[B43] Stimberg M. Brette R. Goodman D. F. (2019). Brian 2, an intuitive and efficient neural simulator. eLife 8:e47314. 10.7554/eLife.47314 31429824 PMC6786860

[B44] Vu T. H. Okuyama Y. Ben Abdallah A. (2019). Comprehensive analytic performance assessment and K-means based multicast routing algorithm and architecture for 3D-NoC of spiking neurons. J. Emerg. Technol. Comput. Syst. 15, 34:1–34:28. 10.1145/3340963

[B45] Waldrop M. M. (2016). More than moore. Nature 530, 144–148. 10.1038/530144a 26863965

[B46] Wu Y. Deng L. Li G. Zhu J. Shi L. (2018). Spatio-temporal backpropagation for training high-performance spiking neural networks. Front. Neurosci. 12:331. 10.3389/fnins.2018.00331 29875621 PMC5974215

[B47] Yin S. Venkataramanaiah S. K. Chen G. K. Krishnamurthy R. Cao Y. Chakrabarti C.. (2017). Algorithm and hardware design of discrete-time spiking neural networks based on back propagation with binary activations, in 2017 IEEE Biomedical Circuits and Systems Conference (BioCAS) (IEEE), 1–5.

[B48] Zamarreño-Ramos C. Linares-Barranco A. Serrano-Gotarredona T. Linares-Barranco B. (2012). Multicasting mesh aer: a scalable assembly approach for reconfigurable neuromorphic structured aer systems. application to convnets. IEEE Trans. Biomed. Circ. Syst. 7, 82–102. 10.1109/TBCAS.2012.2195725 23853282

[B49] Zhao M. Gao B. Tang J. Qian H. Wu H. (2020). Reliability of analog resistive switching memory for neuromorphic computing. Appl. Phys. Rev. 7, 011301. 10.1063/1.5124915

